# Cell Cycle– and Chaperone-Mediated Regulation of H3K56ac Incorporation in Yeast

**DOI:** 10.1371/journal.pgen.1000270

**Published:** 2008-11-21

**Authors:** Tommy Kaplan, Chih Long Liu, Judith A. Erkmann, John Holik, Michael Grunstein, Paul D. Kaufman, Nir Friedman, Oliver J. Rando

**Affiliations:** 1 School of Computer Science and Engineering, The Hebrew University, Jerusalem, Israel; 2 Department of Molecular Genetics and Biotechnology, Faculty of Medicine, The Hebrew University, Jerusalem, Israel; 3 Department of Biochemistry and Molecular Pharmacology, University of Massachusetts Medical School, Worcester, Massachusetts, United States of America; 4 Programs in Gene Function and Expression and Molecular Medicine, University of Massachusetts Medical School, Worcester, Massachusetts, United States of America; 5 Department of Biological Chemistry, Geffen School of Medicine, University of California Los Angeles, Los Angeles, California, United States of America; 6 Molecular Biology Institute, Los Angeles, California, United States of America; Netherlands Cancer Institute, The Netherlands

## Abstract

Acetylation of histone H3 lysine 56 is a covalent modification best known as a mark of newly replicated chromatin, but it has also been linked to replication-independent histone replacement. Here, we measured H3K56ac levels at single-nucleosome resolution in asynchronously growing yeast cultures, as well as in yeast proceeding synchronously through the cell cycle. We developed a quantitative model of H3K56ac kinetics, which shows that H3K56ac is largely explained by the genomic replication timing and the turnover rate of each nucleosome, suggesting that cell cycle profiles of H3K56ac should reveal most first-time nucleosome incorporation events. However, since the deacetylases Hst3/4 prevent use of H3K56ac as a marker for histone deposition during M phase, we also directly measured M phase histone replacement rates. We report a global decrease in turnover rates during M phase and a further specific decrease in turnover at several early origins of replication, which switch from rapidly replaced in G1 phase to stably bound during M phase. Finally, by measuring H3 replacement in yeast deleted for the H3K56 acetyltransferase Rtt109 and its two co-chaperones Asf1 and Vps75, we find evidence that Rtt109 and Asf1 preferentially enhance histone replacement at rapidly replaced nucleosomes, whereas Vps75 appears to inhibit histone turnover at those loci. These results provide a broad perspective on histone replacement/incorporation throughout the cell cycle and suggest that H3K56 acetylation provides a positive-feedback loop by which replacement of a nucleosome enhances subsequent replacement at the same location.

## Author Summary

Wrapping of eukaryotic genomes by the histone proteins impacts virtually all known DNA templates processes. Covalent modification of the histone proteins has emerged as a key mechanism for regulation of transcription and other processes. Here, we report high-resolution genomic mapping of a relatively recently described modification, H3K56 acetylation. We find that H3K56ac is localized at all known sites of nucleosome incorporation, not only during genomic replication, but also during replication-independent nucleosome replacement. By measuring changes in histone replacement during M phase (when K56ac is erased by deacetylases), we find evidence suggesting that histone replacement may help control genomic replication programs. Finally, analysis of histone replacement in mutants in the K56 acetylation pathway provide evidence for self-reinforcing patterns of histone replacement, where replacement of a fresh nucleosome is slower than replacement of a nucleosome that has already been replaced once. These results provide a broad perspective on histone incorporation throughout the cell cycle and deepen our understanding of the relationship between histone turnover and histone modification.

## Introduction

Eukaryotic nuclear genomes are packaged into a nucleoprotein complex known as chromatin, whose repeating subunit, the nucleosome, consists of almost two turns of DNA wrapped around an octamer of histone proteins. The histones are subject to a huge variety of covalent modifications, which have roles in DNA-templated processes ranging from transcription to DNA repair. Identifying the genomic locations of various histone modifications has generated great insight into the mechanisms responsible for deposition of histone marks [1]–[7]. Complementary genetic studies reveal the functions of many of these modifications, which can broadly be separated into those that act via direct biophysical effects on the chromatin fiber [8], and those whose effects are mediated by recruited proteins [9]–[11].

### H3K56 Acetylation

While the best-understood histone modifications are those on the flexible N and C-terminal tails, residues in the core domain are also modified. A recent series of studies has shown that histone H3 lysine 56 (H3K56), in the H3 core domain, is a frequent site of acetylation in fungal species [12]–[15]. Bulk K56 acetylation peaks during S-phase [16],[17], where it plays a role in the DNA damage response [17],[18]. H3K56 is acetylated by Rtt109 [19]–[22], a distant homolog of the p300/CBP histone acetyltransferase [23],[24], and which acts preferentially on free, but not nucleosomal, histones [25]. In vitro, acetylation by Rtt109 is extremely inefficient in the absence of either of two histone chaperone cofactors, Asf1 and Vps75 [25]. Oddly, while Asf1 is required for detectable K56 acetylation in vivo, the major binding partner for Rtt109 in the cell is Vps75, whose deletion has little effect on bulk K56 acetylation levels [25],[26].

### H3K56 Is a Marker for Newly Synthesized Histones during Replication

A variety of data suggests that acetylation of H3K56 is limited to newly synthesized histones, and occurs prior to nucleosomal assembly [17],[25]. Two classes of mechanisms provide for incorporation of new histones into chromatin–replication-coupled (RC) and replication-independent (RI) histone incorporation [27],[28]. During genomic DNA replication, half of the pre-existing maternal histones are transferred to the daughter DNA strands, with the other half-complement of nucleosomes being assembled from newly synthesized histones. During S phase, all newly-synthesized histones are believed to be acetylated at H3K56, and are then deposited throughout the genome. After S phase, H3K56 is deacetylated during G2/M by the Sir2-related HDACs Hst3 and Hst4 [29],[30]. DNA damage down-regulates these HDACs at both the transcriptional and posttranslational levels, allowing K56ac to persist and contribute to the DNA damage response. Accordingly, H3K56R mutants are sensitive to DNA-damaging agents [12],[13],[17],[19],[30],[31]. Recent studies show that H3K56R cells are unable to reassemble chromatin after DNA damage repair [18], and that K56 acetylation promotes association of histones with the CAF-1 and Rtt106 deposition factors, suggesting positive roles for K56 acetylation in the nucleosome assembly process [32].

### Histone Turnover

Alternatively, histones can be replaced via replication-independent histone replacement, or histone turnover [27], [28], [33]–[40]. In metazoans, RI histone replacement utilizes a specific H3 isoform, H3.3 [27],[41]. GFP-H3.3 localizes to highly transcribed genomic loci, and H3.3 appears to be continuously exchanged over transcribed regions as long as they are being transcribed [28],[42]. However, the rate of H3 incorporation during transcription is generally slower than that for the more loosely-associated H2A/H2B dimers. For example, while transcribed regions exhibit high levels of H2A/H2B exchange in the slime mold *Physarum polycephalum*, these regions show much lower levels of H3/H4 turnover [40]. Similarly, recent studies in yeast have found that H2A/H2B exchange rapidly over coding regions [35], while coding region H3 replacement only occurs at relatively high transcription rates [33],[38].

Since RI replacement is observed at locations besides actively transcribed genes, other mechanisms apart from transcription are likely to be involved in replication-independent H3 turnover. This concept is substantiated by genome-wide high-resolution measurements of nucleosome exchange rates. For example, in yeast, rapid histone turnover occurs at nucleosomes over the beginning or end of genes, in contrast to nucleosomes in the middle of ORFs that are replaced much less frequently [33],[35],[38]. Similarly, at Drosophila homeotic loci, where there is relatively little H3.3 due to infrequent transcription, H3.3 is enriched in peaks over regulatory regions [37]. Notably, elevated exchange is observed in both yeast and Drosophila at boundary elements that block the spread of chromatin states [33],[37], suggesting that turnover has a regulatory role in genome organization. Together, these data demonstrate that eukaryotic organisms have mechanisms that promote histone exchange in a locus-specific manner that is unlinked to either DNA replication or gene transcription. Interestingly, recent work revealed a strong correlation between H3K56ac levels and RI histone turnover, and further showed that deletion of *ASF1*, which affects bulk H3K56ac levels, globally slowed H3 replacement [38]. These results suggested that incorporation of K56-acetylated H3 occurs during replication-independent turnover of nucleosomes. However, because the bulk of H3K56ac is synthesized during S phase of the cell cycle, the relative contribution of replication-linked and -unlinked processes, and how they might be regulated at different loci, had not been resolved.

### Cell cycle and Chaperone Regulation of H3K56ac Incorporation

Here, we describe single-nucleosome resolution studies on the genomic distribution of H3K56 acetylation in asynchronously growing “midlog” yeast cultures, as well as in yeast proceeding synchronously through the cell cycle. We find a brief peak of H3K56ac over early-firing replication origins during early S-phase, as expected for DNA replication-coupled deposition. However, these loci exhibit little enrichment of H3K56ac in midlog cultures, consistent with the small fraction of the cell cycle during which H3K56ac is observed at these loci. In contrast to the loci marked by H3K56ac during replication, other loci exhibit high H3K56ac levels in unsynchronized cultures. Such loci are associated with rapid replication-independent nucleosome turnover. Therefore, acetylation of H3K56 marks nucleosomes incorporated via both the replication-coupled and the replication-independent pathways. Additionally, because expression of K56 deacetylases Hst3 and Hst4 during M phase precludes the use of H3K56ac as surrogate readout for histone replacement at this time [29]–[31], we directly characterized H3 replacement during M phase arrest. Notably, several early origins of replication, which exhibited rapid H3 replacement during G1, became “cold” during M phase, suggesting a possible role for H3 replacement in control of origin firing in yeast. Finally, H3K56ac not only marks replication-independent histone replacement, but also appears to enhance this turnover, because direct measurement of H3 replacement rates in mutants lacking the K56 acetylation machinery (Rtt109 and Asf1) revealed diminished H3 replacement. Together, these results provide a dynamic overview of histone incorporation throughout the cell cycle, suggest a biological role for histone replacement in origin firing, and provide evidence for positive feedback in the nucleosome replacement process.

## Results

### Single-Nucleosome Resolution Mapping of H3K56 Acetylation in Yeast Reveals RC and RI Mechanisms for H3K56ac Incorporation

To characterize the genomic distribution of H3K56ac, we used recently-developed techniques to measure chromatin structure at single-nucleosome resolution [5]. We first measured the H3K56 acetylation levels per nucleosome in unsynchronized midlog cultures. Briefly, midlog yeast cultures were fixed with formaldehyde, chromatin was digested to ∼80% mononucleosomes, and DNA associated with H3K56ac was immunoprecipitated. H3K56ac IP was labeled and hybridized competitively with labeled mononucleosomal “input” DNA, yielding H3K56ac levels per nucleosome (i.e. controlled for histone abundance) across chromosome 3, and ∼230 additional loci (4% of the genome).

**Figure 1:**
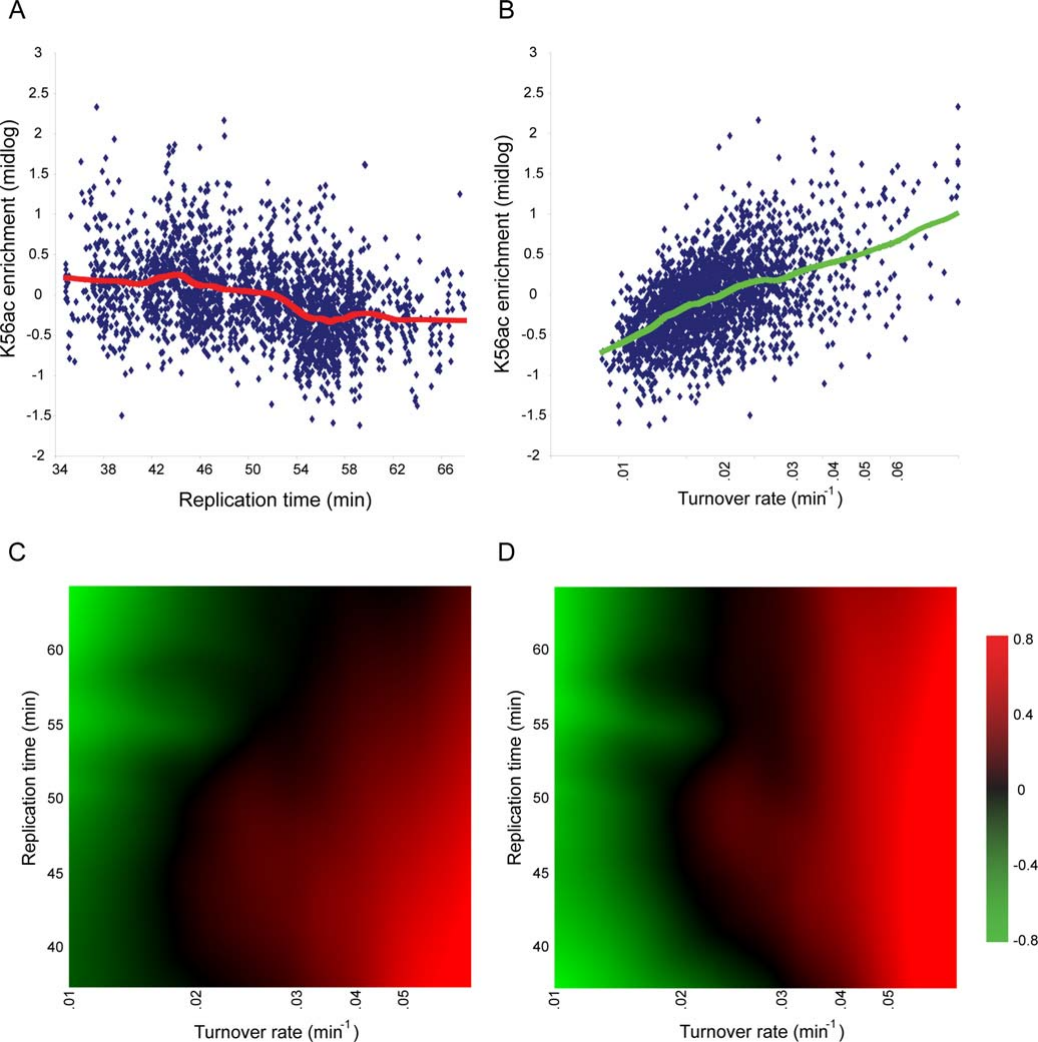
H3K56 Acetylation levels are partially explained by genomic replication timing, RI turnover rates, or both. (A) Scatter plot of the midlog H3K56ac for each nucleosome vs. its replication timing [43],[44]. Red line shows a LOWESS-based smoothing of the H3K56ac levels, explaining 17.3% of the variance in H3K56ac levels. (B) Same as (A), but for replication-independent histone replacement rates during G1-arrest [33]. Here, LOWESS-based fit (green) explains 31.4% of the variance in H3K56ac levels. (C) Heatmap visualization of the 3D LOWESS-based fit of H3K56ac levels, based on both RI turnover rates (X-axis) and replication timing (Y-axis). Here, 38% of the variance in H3K56ac levels are explained. (D) Same as (C), but for H3K56ac levels in G1-arrested cells.

Given the well-established link between H3K56ac and replication, we first compared midlog H3K56ac levels to genome-wide replication time measurements [43],[44]. Previous studies [17],[29] showed H3K56ac in newly synthesized histones deposited during S phase. If this were the only mechanism for H3K56ac incorporation, then early-replicating regions would carry acetylated H3K56 for the greatest fraction of the cell cycle before erasure by Hst3/4 during G2/M phase [17],[29], and would display high midlog K56ac levels. However, as Figure 1A shows, only a limited fraction (17.3%) of the variance in midlog acetylation can be explained by replication-coupled K56ac incorporation (Methods). Instead, the majority of genomic loci enriched for H3K56ac during asynchronous growth were not found adjacent to origins of replication. We therefore conclude that replication timing plays a minor role in the overall level of H3K56ac on any given nucleosome, in agreement with the relatively short period from early S to M cell cycle phases. These data suggest involvement of additional mechanisms for H3K56ac incorporation, corresponding either to its escape from M phase erasure, or incorporation prior to S phase.

Tsubota *et al.* showed the H3K56 acetyltransferase Rtt109 can acetylate free (but not nucleosomal) histones [25], suggesting that H3K56ac association with a genomic locus must result from a nucleosome incorporation event, rather than acetylation in situ. We therefore considered replication-independent (RI) histone replacement as an additional contributor to H3K56ac levels in the midlog measurement. Indeed, Rufiange *et al.* recently found significant correlations between H3 turnover and H3K56ac levels in midlog cultures [38]. We compared H3K56ac levels to data from a recent study in which we used an inducible epitope-tagged histone to measure rates of replication-independent histone replacement during G1-arrest [33]. As shown in Figure 1B, the midlog acetylation levels of H3K56 are significantly associated with replication-independent turnover rates, which account for 31% of variance in midlog H3K56 acetylation levels. Grouping nucleosomes by their locations relative to underlying genomic features [5] showed that H3K56ac is enriched during midlog growth at the same classes of loci (promoters, tRNA genes) that were found to show rapid replication-independent nucleosome exchange (Figure S1).

Neither replication times nor turnover rates perfectly account for the midlog levels of H3K56 acetylation. Part of this is due to the difference between experimental protocols for these measures–G1 turnover rates were measured in alpha factor-arrested yeast, and alpha factor-inducible genes such as *FUS1* exhibit more rapid turnover during this arrest than they do during midlog growth [33], but midlog H3K56ac levels here were measured in unperturbed cells. Despite these experimental differences, much of the variability (∼38%) in H3K56ac levels can be captured using replication and turnover together–Figure 1C shows midlog K56ac levels as a function of both replication time and replication-independent turnover rate. Notable is the observation that, except at very early replication times or for very rapid turnover, the K56ac level at a nucleosome is independently controlled by these two processes, as evidenced by the dominant diagonal visible here and also easily visualized in topographic representations of the data (Figure S2A).

To experimentally separate contributions to K56 acetylation due to replication vs. turnover, we arrested yeast in G1 and repeated our K56 acetylation measurement, removing replication timing as a confounding factor. As predicted, K56 acetylation levels in this data were still correlated with nucleosome turnover rates, but no longer exhibited any relationship to replication times (Figures 1D and S2B).

### Cell Cycle Variation in H3K56ac Deposition

**Figure 2:**
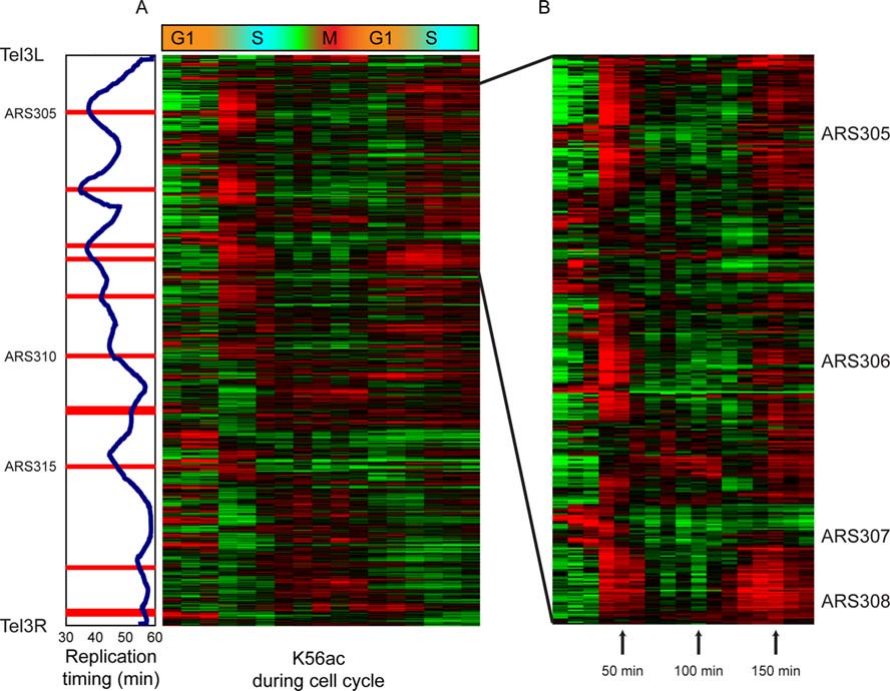
Cell cycle profile of H3K56ac levels. (A) Shown are H3K56ac enrichments for every nucleosome on chromosome 3, sorted by genomic location from Tel3L (top) to Tel3R (bottom). Vertical panel on left shows the genomic replication timing for each nucleosome [43],[44], and the position of origins of replication (ARS) [59]. (B) Zoom over the genomic region of early firing origins ARS305-ARS308.

The identification of H3K56ac as a marker for both replication-coupled and replication-independent histone incorporation suggests that every time a nucleosome is inserted into the genome it carries in H3K56ac. Therefore, we performed genome-wide analysis of H3K56ac incorporation during cell cycle progression as a marker of sites of new histone incorporation. Cultures of *cdc28-13* yeast were arrested in G1 phase at the restrictive temperature, then released into the cell cycle by rapid cooling to the permissive temperature by mixing with cold culture media. Over the course of 170 minutes (∼1.5 cell cycles), culture samples were fixed every ten minutes with formaldehyde, and H3K56ac levels at individual nucleosomes were measured relative to the cell cycle average. This experiment measures variation in H3K56ac levels throughout the cell cycle, allowing comparison to the average midlog enrichment at the locus in question. As a control for total histone occupancy at each locus, we hybridized bulk nucleosomal DNA in a parallel series of experiments. Finally, we isolated mRNA from each time point, and hybridized it to gene expression microarrays as an internal control for cell cycle synchrony. mRNA abundance measures confirmed good cell cycle synchrony, and provided us with cell cycle phase information for each sample (Figure S3). Figure 2A shows H3K56ac enrichment for every nucleosome on chromosome 3 through the cell cycle, sorted by genomic location.

Immediately apparent on inspection of the H3K56ac cell cycle data are several peaks of H3K56ac that occur at early S-phase, about ∼40–50 minutes after release from G1. In total, the genome contains ∼100 such nucleosomes, which are generally centered over early origins of replication. Several examples of H3K56ac incorporation overlapping early origins are highlighted in Figure 2B. We interpret these peaks as regions where H3K56ac was incorporated into chromatin during early S phase, as replication begins. These peaks disappear at ∼60 minutes, coincident with uniform, genome-wide presence of K56-acetylated histones by the end of S phase, with resulting loss of the *relative* enrichment observed at early origins at the beginning of S phase. Later, H3K56ac levels are erased by M phase expression of Hst3/4, around 100 minutes after release. Early S phase peaks were not artifacts of changes in histone occupancy (data not shown). To test the generality of the anecdotal examples shown in Figures 2A and 2B, we aligned nucleosomes by their replication times [43],[44]. Clear in these alignments is the 40–50 minute peak of H3K56ac at early-replicating regions of the genome (Figure S4A), showing that the early S phase peaks of H3K56ac over early-replicating regions are general.

As described above, midlog levels of H3K56ac do not correlate particularly well with replication timing. Indeed, comparison of cell cycle H3K56ac data to midlog enrichments revealed that many early-replicating regions (i.e. regions near early ARSs, though not the ARSs themselves–see below) exhibited relative depletion of H3K56ac in unsynchronized cultures (Figure S5). This phenomenon holds despite clear S phase H3K56ac peaks (e.g. around ARS305, Figure S5B), and is consistent with the interpretation that H3K56ac is only found on these nucleosomes during a brief period of the cell cycle, from early S phase until M phase.

In contrast, as shown in Figure 1B, replication-independent nucleosome exchange rates strongly correlate with midlog H3K56ac levels. At rapidly-replaced nucleosomes, inspection of the H3K56ac distribution during the cell cycle reveals a distinctive behavior. Typically, these “hot” nucleosomes exhibit high levels of H3K56ac starting in G1 (Figure S5C). This phenomenon is further emphasized when cell cycle K56ac profiles were sorted by the relevant midlog turnover rates (Figure S4B). We interpret these data as evidence that H3K56ac is incorporated on hot nucleosomes when they are replaced, and this is most clearly observed during G1 phase (following M phase K56 deacetylation). Thus, the longer time period from G1 to M phase results in increased midlog enrichment of H3K56ac at hot nucleosomes relative to cold nucleosomes that replicate early, because the latter nucleosomes only carry H3K56ac from S until M.

### From Log Ratios to Absolute Acetylation Percentages

To disentangle the quantitative contributions of replication and turnover to H3K56ac levels, we decided to use the relative measurement of H3K56ac log ratios during the cell cycle to generate an estimate of the absolute levels of H3K56 acetylation per nucleosome. This approach was motivated by a desire to estimate the absolute acetylation levels for each nucleosome over time, thus “fixing” microarray-related artifacts, including quenching and saturation at high or low values, different normalization values along the cell cycle (due to change in overall H3K56 levels), as well as loss of cell cycle synchronization. For each nucleosome, our procedure finds the absolute acetylation levels that best explain several observations, including midlog and cell cycle levels of H3K56ac, cell cycle microarray ratios, and the abundance of bulk H3K56ac as reflected in the amount of DNA isolated from the immunoprecipitated nucleosomes (Methods).

First, we analyzed midlog H3K56ac data. By reverse-engineering the hybridization pre-processing and measurements, we were able to estimate, for each genomic nucleosome position, the percentage of cells in which it was acetylated at H3K56. Briefly, these absolute values were calculated by exponentiating the array measurements (log_2_ H3K56Ac levels vs nucleosomal occupancy). These numbers were then linearly transformed to 0–100% scale, to correct for array normalization (Figure S6, Methods). The majority of nucleosomes were acetylated in relatively few cells (<40%), consistent with the short duration of S phase in yeast, and with the low median rate of nucleosome replacement [33]. Moreover, by averaging the H3K56ac absolute acetylation levels (over all measured nucleosomes) we estimated that 31% of the bulk population of histone H3 is acetylated during midlog (Figure S6). This agrees well with independent mass spectrometric data showing that 28% of H3K56 is acetylated in midlog yeast [15].

We then applied the same approach to convert array-based log ratios (H3K56ac at specific time vs. H3K56ac at mid-log) into absolute acetylation levels along the cell cycle. A complicating factor is that a global reduction in total H3K56ac (*e.g.*, at M phase) in the culture artificially increases measured ratios. Since such global shifts exist we cannot use standard normalization methods. Instead, we estimate array-specific bias factors based on the amount of DNA isolated from immunoprecipitated nucleosomes (Methods). We then generate our quantitative estimations of H3K56 acetylation along the cell cycle, by combining the estimate of absolute H3K56ac levels from midlog cells and these array-specific factors (Figure S7, Methods).

**Figure 3:**
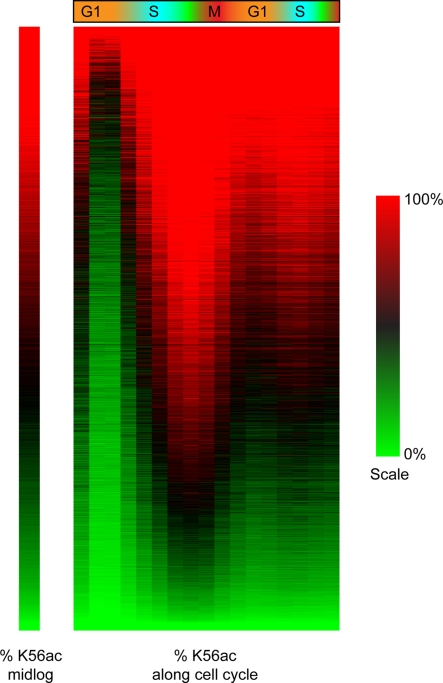
Absolute H3K56ac levels. Absolute percentages of H3K56 acetylation were calculated based on midlog H3K56 levels (left) and time-specific abundance of bulk H3K56 acetylation, as reflected in the amount of DNA isolated from the immunoprecipitated nucleosomes (Methods). Columns indicate 10 minute intervals. The nucleosomes are sorted by their midlog H3K56ac levels. Note that the difference between the zero and ten minute time points likely reflects the fact that the zero time point represents data from yeast still arrested in G1 at 37 degrees.

Figure 3 shows the estimated % K56 acetylation for nucleosomes as a function of cell cycle, with nucleosomes aligned by their midlog H3K56ac log ratios. Notable here is the pair of peaks associated with completed S phase, when most nucleosomes are at least 50% acetylated on H3K56. Furthermore, hot nucleosomes exhibit high levels of H3K56ac throughout the cell cycle (Dataset S1). H3K56 acetylation is correlated both with replication times during S phase, and with G1 rates of turnover, two processes that result in histone incorporation into the genome (Figures 1 and 2).

### Kinetic Model of H3K56ac Based on Replication Timing and Turnover Rate

To ask whether replication times and turnover completely explain H3K56ac profiles along the cell cycle, and in particular to determine whether there is evidence for an additional phase of H3K56ac incorporation, we developed a kinetic model of H3K56ac. Our model simulates nucleosome incorporation and H3K56 deacetylation as a non–homogenous Poisson process, resulting with a system of time-dependent rate equations (Figure S8, Methods). This model is based on three parameters for each nucleosome, including replication timing (experimentally measured by [43],[44]), RI turnover rates (measured by [33]), and initial H3K56ac levels in G1-arrested cells. In addition, the model depends on additional global parameters (shared for all genomic loci), including estimations of the time-dependent activity of the deacetylases Hst3/4. Put together, the model dictates for each locus when during the cell cycle, and at what rate, a new H3K56 acetylated nucleosome will be deposited (either due to a replication-coupled or a replication-independent process), and the rates at which acetylated nucleosomes will be deacetylated. For each time point, our quantitative model outputs the percent of nucleosomes acetylated at any locus. Finally, we optimized the global- and nucleosome-specific parameters of the model, to minimize its deviation from measured turnover rates, replication times, as well as midlog and cell cycle H3K56ac levels (Methods).

**Figure 4:**
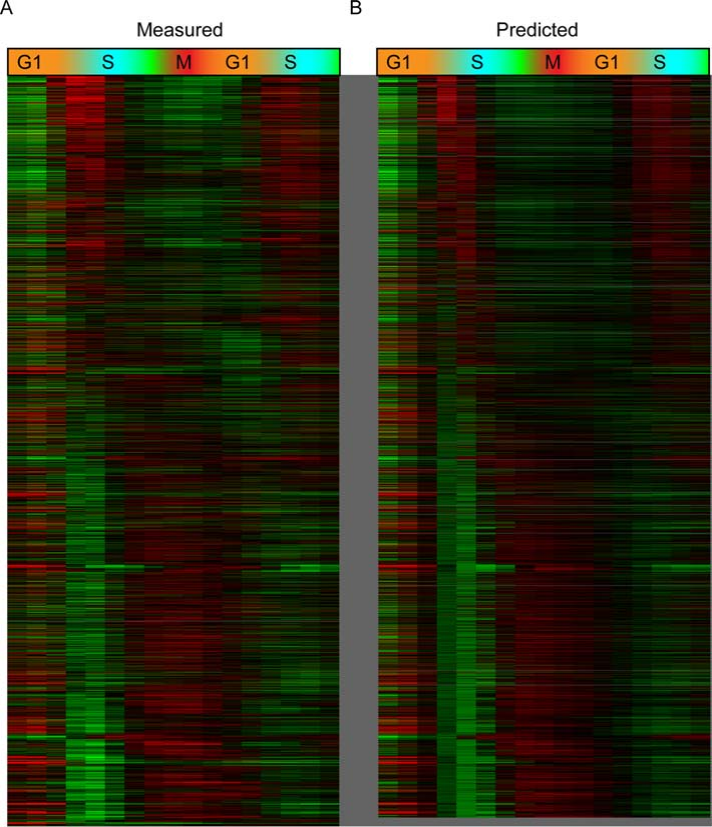
Cell cycle H3K56ac profiles modeled by replication times and replication-independent turnover rates. (A) Measured microarray log ratios of H3K56ac along the cell cycle, sorted by replication times, from early (top) to late (bottom). (B) Predicted H3K56ac ratios were estimated using a kinetic model H3K56ac levels (Methods). In brief, our model simulates the percent H3K56ac for each nucleosome along time, depending on S phase replication time (accompanied by genomic integration of H3K56ac nucleosome), replication-independent nucleosome turnover (by H3K56ac nucleosomes), and time-specific activity levels of the deacetylases Hst3/4. Quantitatively, our model predictions explain 66% of the variance presented by the measured data (left).

Figure 4 shows the measured microarray log ratios of H3K56ac along the cell cycle, compared to predictions made by our quantitative model. Here, we optimized two parameters for each nucleosome–the acetylation levels in G1-arrested cells were estimated based on t = 0 measurements (Figure 1D), and the exact S phase timing of replication [43],[44] was fine-tuned to fit the cell cycle data to account for potential variation in the kinetics of cell cycle re-entry after arrest (Figure S9). Quantitatively, the H3K56ac levels predicted by our rate model explain 66% of the variance presented by the measured data. The excellent agreement between the measured and predicted ratios shows that S phase replication times and RI turnover rates capture much of cellular K56ac physiology. This suggests that G1 turnover and S phase genomic replication together capture the bulk of “first-time” H3 incorporation events in a given cell cycle.

### M phase Turnover Rates

However, it is important to note that cell cycle profiling of H3K56ac can only identify the *initial* K56ac incorporation event in a given cell cycle–replacement of a K56-acetylated nucleosome with another acetylated nucleosome is of course invisible in this assay. Furthermore, even if H3K56ac profiling across the cell cycle could in principle provide information about any nucleosome incorporation event, M phase turnover events are largely “invisible” due to high activity levels of the H3K56 deacetylases Hst3/4 [29]–[31]. We therefore decided to directly explore the dynamics of nucleosome turnover during M phase. For this, we arrested yeast carrying a galactose-inducible Flag-tagged copy of H3 at M phase using benomyl/nocodazole. We then induced synthesis of Flag-tagged histones during M phase arrest, and measured the position-specific Flag/Nuc ratio at various time points after Gal induction, using chromatin immunoprecipitation followed by hybridization to high-resolution tiling microarrays. We analyzed the kinetics of these ratios using a simple rate model, similar to the one described previously [33], and estimated nucleosome-specific semi-quantitative M phase turnover rates (Dataset S2, Methods). This approach was validated by revisiting the G1 phase turnover rates. We compared our previous G1 rates (by Flag/Myc ratios [33]) to new G1 Flag/Nuc-based rates (i.e. no Myc tag), yielding similar results (R = 0.70, p<1e-300, Table S1).

**Figure 5:**
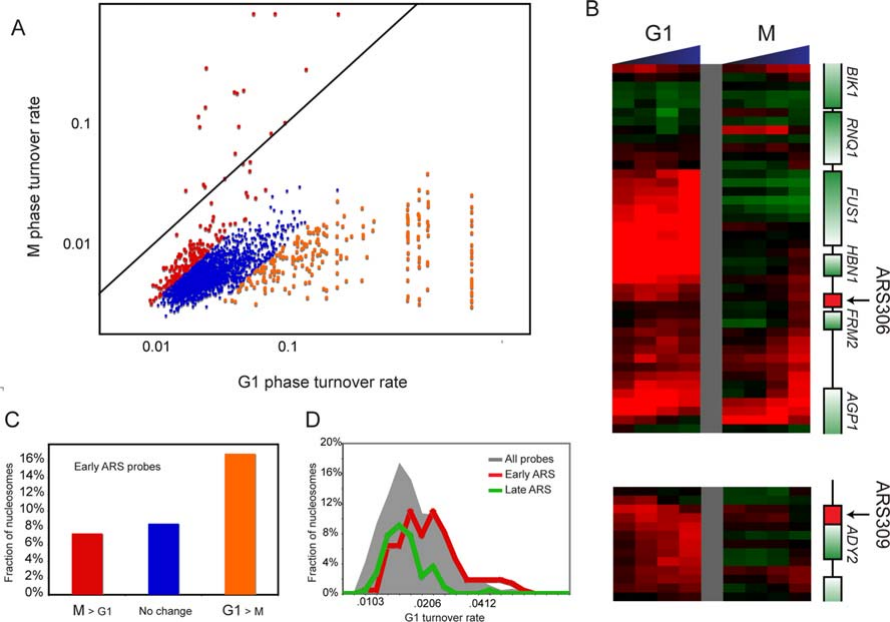
G1 vs M phase turnover rates. (A) Comparison of replication-independent nucleosome turnover rates during G1 (X-axis) and M phase arrest (Spearman rank correlation coefficient of 0.66). Nucleosomes with differential turnover rates are highlighted orange (more rapid during G1) or red (more rapid during M). Solid black diagonal line represents the Y = X line, showing a 5-fold global decrease in M phase turnover rates relative to G1 phase rates. (B) Two genomic loci exhibiting rapid H3 turnover during G1 phase (left), that dramatically switch to slow turnover rates during M phase (right). Shown are the *FUS1* genomic locus and the early origin or replication ARS306 (top) and the origin of replication ARS309 (bottom). (C) Nucleosomes around early firing origins of replication are significantly enriched among nucleosomes with rapid G1 (but not M) replication-independent turnover rates (two-fold enrichment, HyGe p<7.3e-5). (D) Nucleosomes surrounding early firing origins of replication (red) are replaced rapidly during G1, as compared to late firing loci (green), and to the background distribution of all nucleosomes (grey).

In general, replication-independent turnover rates during M phase are highly-correlated (R = 0.63, p<2e-251, Table S1) with replication-independent turnover rates during G1 phase, though there appears to be ∼5-fold global slowing of H3 replacement during M phase (Figure 5A). While this does not seem to be an artifact of slower induction of pGal-Flag-H3 in M phase (data not shown), we urge caution in using this assay to measure *bulk* turnover rates, as this can be done with greater temporal precision using FRAP. Instead, our assay is specifically tailored to measuring the *relative* variation in H3 replacement rates (among various genomic loci). However, specific changes in turnover were observed layered on top of the general M phase slowdown.

Notably, a handful of loci that exhibit rapid H3 turnover during G1 phase dramatically switch to slow turnover rates during M phase (Figure 5B). Many of these loci correspond to genes (*e.g.*, *FUS1*, Figure 5B) induced by the alpha factor used to arrest cells in G1, which also induces a transcriptional reprogramming that leads to changed histone turnover at a number of loci [33]. Beyond alpha factor-responsive genes, many of the nucleosomes that “cool” during M phase correspond to early origins of replication. As Figure 5C shows, nucleosomes near early firing origins are enriched approximately two-fold among nucleosomes that exchange more rapidly in G1 than in M (p<7.3e^−5^). Because we observed high levels of midlog H3K56 acetylation and rapid G1 turnover at nucleosomes near early ARS elements (Methods, Figure 5D), these data suggest that high turnover in G1 is important for early firing upon entry into S. Additionally, our data suggest that a major qualitative change in nucleosome dynamics between G1 and M phase is a slowing of nucleosome exchange over early ARS sequences, which we hypothesize may play a mechanistic role in preventing re-replication at origins of replication ([45], Discussion). Other changes in turnover between G1 and M phase are largely related to the use of alpha actor during G1, but additional changes do occur whose biology is not immediately apparent despite enriched features (such as noisy expression) from other genomic datasets (Dataset S3).

### Role of Histone Chaperones in K56 Acetylation

Finally, the correlation between H3K56ac and replication-independent histone replacement leads us to a typical conundrum in the chromatin mapping field–when a mark is deposited during some process, is it required for that process or for some subsequent pathway? In other words, does H3K56ac simply mark RI histone turnover, or does it contribute to the efficiency of turnover? To address these questions, we measured H3 turnover in mutant strains lacking subunits of the H3K56 acetylation machinery, using galactose induction of Flag-H3 in G1-arrested cells, followed by Flag immunoprecipitation to identify loci experiencing H3 replacement.

The K56 acetyltransferase Rtt109 requires a histone chaperone to function efficiently in vitro and in vivo [25]. Two chaperones stimulate the acetyltransferase activity of Rtt109: Asf1, which plays a role in a wide range of histone deposition and replacement events in the cell, and Vps75, a NAP1-like protein whose *in vivo* function is obscure [25]. Deletion of Asf1 abolishes detectable K56ac in vivo [26]; in contrast, Vps75 is the more abundant binding partner for Rtt109 in the cell [46], but is important for bulk H3-K9 and H3-K23 acetylation rather than H3-K56 acetylation *in vivo* [47],[48]. We therefore constructed strains with deletion of one of *RTT109*, *ASF1*, and *VPS75*, and measured histone replacement in these strains.

**Figure 6:**
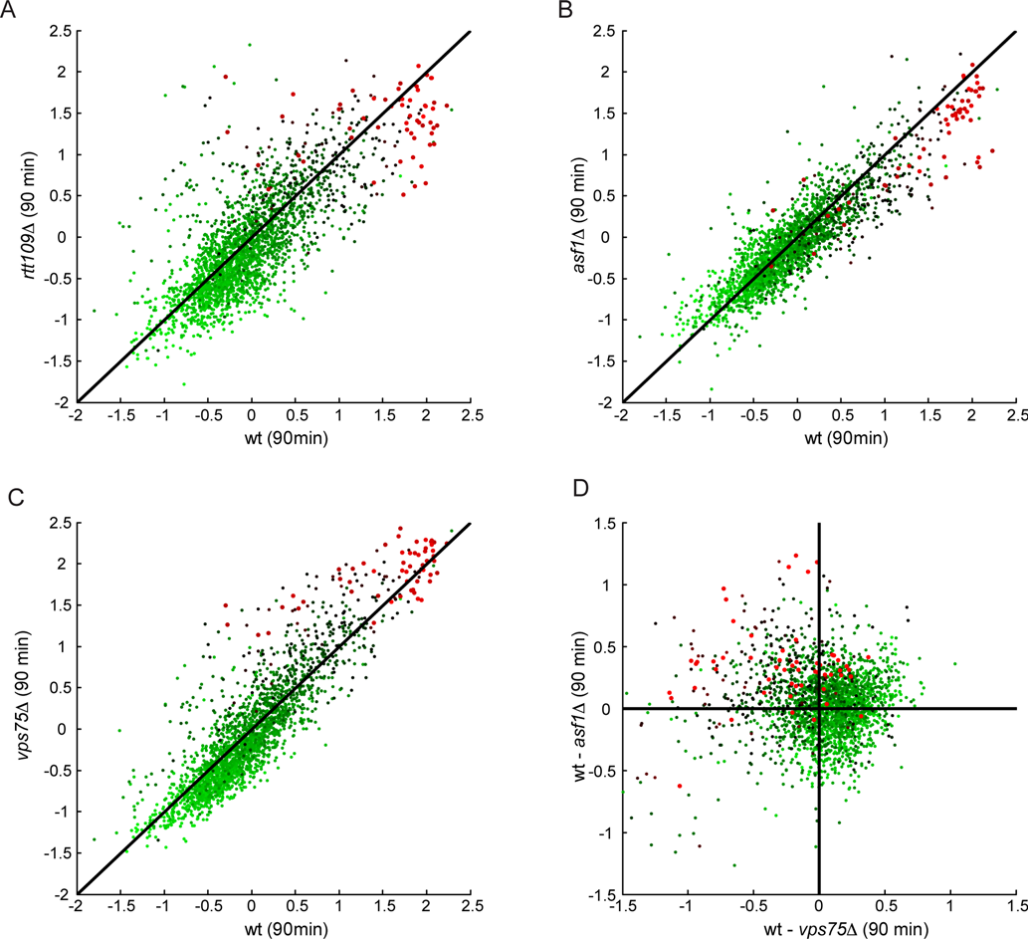
H3K56ac machinery and histone turnover. (A–C) Comparison of replication-independent nucleosome turnover during G1 for wild-type yeast (X-axis, expressed as log_2_ ratio of Flag/nucleosome at 90 minutes of Flag-H3 induction), and mutant yeast (Y-axis). Comparisons are shown for *rtt109*Δ (A), *asf1*Δ. (B), *vps7*Δ (C). Dots are colored by G1 turnover rates from Dion et al., with red dots indicating hot nucleosomes. X = Y diagonal is included to indicate unchanged turnover rates. Note that hot nucleosomes fall to the right of X = Y in (A) and (B), indicating that they cool down in these mutants, while hot nucleosomes actually are replaced more rapidly in *vps75*Δ. (D) Asf1 and Vps75 have opposing effects on histone replacement. Deviation between wild-type and the indicated mutant are sown, with nucleosomes color-coded as in remaining panels. Red dots in upper-left quadrant indicate nucleosomes that slow turnover in *asf1*Δ, but speed up in *vps75*Δ.

Deletion of Rtt109 and Asf1, both of which eliminate detectable bulk cellular H3K56ac [20],[22],[26], resulted in slowed H3 replacement at hot nucleosomes (as indicated by red dots to the right of X = Y diagonal in Figure 6A,B). However, these data are complicated by the observation that Flag-H3 was induced more slowly in these strains than in wild-type (not shown). Nonetheless, the slowed histone turnover in our data is consistent with results from Rufiange *et al.*, who found H3 replacement diminished in *asf1*Δ cells, but did not observe reduced kinetics of Flag-H3 induction in the yeast strain used for that study [38]. Moreover, hot nucleosomes preferentially slow down more than “lukewarm” nucleosomes in *rtt109*Δ and *asf1*Δ, again suggesting that these results result from a specific mutant effect on very rapid turnover rather than an artifact of lower Flag-H3 induction levels.

Importantly, in our data we observed that loci which are “hot” in wild-type cells are generally those that continue to be most rapidly replaced in *asf1*Δ cells, with elimination of H3K56ac simply slowing the exchange rate, but not the identity, of rapidly-replaced loci within each strain. Together, these data support the idea that K56ac stimulates histone replacement, consistent with its globular position at the DNA entry point [49, Ozdemir, 2005 #672], and with recent studies indicating that this modification is important for disassembly events during transcription, and assembly events during DNA repair [18],[50]. Conversely, deletion of *VPS75* resulted in higher Flag/Nuc ratios at hot nucleosomes (Figure 6C), suggesting that in wild-type cells Vps75 either slows turnover rates or stabilizes a subset of nucleosomes at hot loci. To determine whether Vps75 and Asf1 had opposing effects on nucleosome replacement, we plotted deviation from wild-type behavior in each mutant against the other mutant (Figure 6D). Hot nucleosomes were concentrated in one quadrant of the scatterplot, showing a relative decrease in Flag-H3 incorporation in *asf1*Δ cells, mirrored by an increase in Flag-H3 incorporation in *vps75*Δ cells. These results support a model in which Vps75 and Asf1 contribute to distinct pathways for histone metabolism in the cell, although the subtle effects observed demonstrate that K56 acetylation is not the major driver of histone replacement in the cell. One possibility is that loss of Vps75 enhances flux through the Asf1/replacement pathway. Alternatively, the different H3 lysine specificities of the Rtt109-Asf1 and Rtt109-Vps75 enzyme complexes [47],[48] may have differential effects on histone turnover.

## Discussion

### K56 Acetylation Marks All Known Histone Incorporation Events during the Cell Cycle

We provide evidence from midlog measurements of H3K56ac levels that this modification marks nucleosomes incorporated into the genome via both replication-coupled and replication-independent mechanisms (See Figure S10 and Table S1 for characterization of antibody specificity). From a technical standpoint, these results provide a demonstration that midlog chromatin measurements may obscure transient events of great significance. Previously, midlog H3K56ac mapping had identified nucleosome replacement as the major contributor to K56ac incorporation [22],[38], while genetic and biochemical studies had emphasized an S phase role for this modification [17],[19]. Since most microarray studies only show *relative* enrichment and depletion of the factor being measured (mRNA, transcription factor levels, etc.), midlog measures of K56ac miss its key role in S phase since it appears to be everywhere and this very uniformity precludes observation of relative enrichment. Instead, the rare nucleosomes that carry K56ac due to turnover in G1 contribute disproportionately to the microarray signal because the time from S to the M phase erasure is shorter than the time from G1 to M.

We therefore measured H3K56ac levels in synchronized cultures throughout the cell cycle, demonstrating that both replication and exchange make large, independent contributions to H3K56ac biology. The synchronized cell data provide a window into histone incorporation throughout all of the cell cycle except M phase, which is obscured by high activity levels of the H3K56 deacetylases Hst3/4. By modeling absolute levels of acetylation on H3K56, we did not find evidence for an additional major phase of histone incorporation. Intriguingly, estimated H3K56ac levels for hot nucleosomes are near 100% throughout the cell cycle. Given that H3K56ac is believed to serve as a mark for newly-replicated chromatin during S phase and on newly repaired chromatin [17],[18], this raises the question of what biological signal is transmitted by H3K56ac at other stages of the cell cycle or in the absence of DNA damage.

### Altered Histone Turnover during M Phase

The tight coupling between H3K56ac incorporation and histone deposition suggests that our cell cycle profiling should capture every “first time” nucleosome incorporation event in the genome, except during M phase. We therefore measured histone exchange during M phase arrest, discovering slowing of nucleosome replacement at several origins of replication (Figure 5B). It is important to note that the slowdown between G1 and M phase could occur at any intervening time period, and indeed we would predict that early ARSs exhibit slowed exchange by mid-S phase after they have fired. Because several studies indicate that the positioning of nearby nucleosomes affects origin function [51]–[53] we hypothesize that this slowed exchange may contribute to the establishment of a non-permissive chromatin structure after origin firing, thereby helping to enforce the requirement that origins fire only once per cell cycle (reviewed in [45]). Furthermore, histone modifications are also known to affect origin firing [54],[55]. Because the Rtt109-Asf1 and Rtt109-Vps75 complexes favor different histone H3 lysine substrates for acetylation [47],[48], this specificity may provide a further means of altering turnover of specific nucleosomes. Therefore, future studies will be required to determine which of these mechanisms may be responsible for the observed M phase regulation at origins.

### Role of K56 Acetylation in Histone Turnover

Finally, our data provide further evidence that H3K56ac not only marks “fresh” nucleosomes, but in fact contributes to the histone replacement process. We note that this contribution could occur either by speeding turnover, or by enhancing the use of free histones during histone incorporation rather than re-use of old nucleosomes in cis, which in our epitope induction assay are indistinguishable. Deletion of *ASF1* or *RTT109* both slowed turnover at hot nucleosomes, and while the effects of these deletions on Flag-H3 induction require that these results be treated with caution, aspects of our data (specific slowing of hot nucleosomes rather than global decrease in Flag/nucleosome ratios), and independent confirmation from other laboratories which do not use epitope induction systems [50],[56] or with no reported defect in epitope induction [38] give us some confidence in the result.

Importantly, in wild-type, *asf1*Δ and *rtt109*Δ cells the same loci remain relatively hot, suggesting that H3K56ac is not required for histone replacement but rather aids the process. What, then, is the role for H3K56ac in histone turnover? Recent data using the inducible *PHO5* model gene demonstrate that histone eviction is slowed in H3K56R mutants [50], suggesting that H3K56ac can aid histone eviction, in addition to potentially aiding histone deposition [18],[32]. These results suggest a positive feedback loop in histone replacement. After M phase, the first nucleosome evicted at any given location is H3K56deac, and predicted to be relatively difficult to evict. The first round of replacement results in an H3K56ac nucleosome, which would subsequently be more amenable to eviction. This self-reinforcing mechanism has similarities to models for transcription in which an initial “pioneer” round of polymerase elongation enhances the probability of subsequent rounds [57].

Two classes of potential mechanisms could account for this positive feedback between turnover and H3K56ac. First of all, K56 is located near the DNA entry-exit points on the nucleosome [13],[49], suggesting that neutralization of the positive charge by acetylation could biophysically alter the nucleosome. Consistent with this idea, nucleosomes containing a H3K56Q alteration display ∼2× faster rates of nucleosome sliding *in vitro* [58]. Also, chromatin in H3K56Q mutants is more accessible to nucleases [17], again consistent with “opening” of chromatin by this modification. Alternative to models centered on structural effects, the H3K56 acetylation may instead serve as a recruitment site for trans-acting factors. For example, preventing H3K56 acetylation inhibited recruitment of the SWI/SNF ATP-dependent chromatin remodeler to histone gene promoters [15], suggesting that eviction could be aided by or required for recruitment of nucleosome-mobilizing complexes in a K56ac-dependent manner. These two classes of model are not mutually exclusive, and one or the other could be predominant at different genomic loci.

These results provide a dynamic perspective on histone replacement throughout the cell cycle, and show that H3K56ac not only marks histone incorporation but also can enhance the replacement process.

## Materials and Methods

### Yeast Culture

18 flasks each of 225 mL BY4741 *cdc28-1^ts^* cells were grown in YPD to an A_600_ OD of 0.45 in 2 L baffled flasks shaking at 200 rpm at room temperature (22°C). The flasks were transferred to a 37°C incubator and incubated, shaking at 200 rpm for 3 hours to arrest and synchronize the cells. To release from arrest, 225 mL 12°C YPD was added to each flask and then returned to the room temperature shaker. Upon release, time points were collected at 10 minute intervals, beginning with t = 0 minutes, with each flask of 450 mL culture representing a time point. For each time point, 37% formaldehyde was added to a 1% final concentration, and the cells were incubated for 15 minutes at room temperature, shaking, at 200 rpm. 2.5 M glycine was added to a final concentration of 125 mM to quench the formaldehyde. The cells were transferred to a 500 mL centrifuge jar on ice and then let stand until groups of 6 time points had accumulated. The cells were spun down at 3000×g for 5 minutes at 4°C and washed once with 50 mL of room temperature MilliQ water. Subsequent procedures were performed in batches of 6 time points.

### Micrococcal Nuclease Digestion

The cell pellets were resuspended in 39 ml Buffer Z (1 M sorbitol, 50 mM Tris-Cl pH 7.4), 28 µl of β-ME (14.3 M, final conc. 10 mM) was added, and cells vortexed to resuspend. 1 ml of zymolyase solution (10 mg/ml in Buffer Z; Seikagaku America) was added, and the cells were incubated at 28°C shaking at 200 rpm, in 50 ml concial tubes, to digest cell walls. Spheroplasts were then spun at 3000×g, 10 min, 4°C. Spheroplast pellets were resuspended and split into aliquots of 600 µl of NP-S buffer (0.5 mM spermidine, 1 mM β-ME, 0.075% NP-40, 50 mM NaCl, 10 mM Tris pH 7.4, 5 mM MgCl_2_, 1 mM CaCl_2_) per 90 ml cell culture equivalent. 40 units of micrococcal nuclease (Worthington Biochemical) were added, and the spheroplasts were incubated at 37°C for 20 minutes–this was determined in initial titrations to yield >80% mononucleosomal DNA, but to repeat these results an independent titration should be carried out as a preliminary study. The digestion was halted by shifting the reactions to 4°C and adding 0.5 M EDTA to a final concentration of 10 mM.

### Chromatin Immunoprecipitation

All steps were done at 4°C unless otherwise indicated. All IPs were processed in parallel. For each aliquot, Buffer L (50 mM Hepes-KOH pH 7.5, 140 mM NaCl, 1 mM EDTA, 1% Triton X-100, 0.1% sodium deoxycholate) components were added from concentrated stocks (10–20×) for a total volume of 800 µl per aliquot. Each aliquot was incubated with 80–100 µl 50% Sepharose Protein A Fast-Flow bead slurry (Sigma) equilibriated in Buffer L for 1 hour on a tube rotisserie rotator. The beads were pelleted with a 1 minute spin at 3000×g, and approximately 2.5–5% of the supernatant for each time point was set aside as ChIP input material. With the remainder, 7 µl anti-H3K56Ac antibody (Upstate #07-677) was added to each aliquot, corresponding to 20% of a 450 ml cell culture.

These were incubated, rotating, overnight (∼16 hours), after which the sample was transferred to a tube containing 80–100 µl of 50% Protein A bead slurry. The sample was incubated with the beads for 1–4 hours for the IP, after which the beads were pelleted by a 1 minute spin at 3,000×g. After removal of the supernatant, the beads were washed with a series of buffers in the following manner: 1 ml of the buffer would be added, and the sample rotated on the tube rotisserie for 5 minutes, after which the beads would be pelleted in a 30 second spin at 3,000×g and the supernatant removed. The washes were performed twice for each buffer in the following order: Buffer L, Buffer W1 (Buffer L with 500 mM NaCl), Buffer W2 (10 mM Tris-HCl pH 8.0, 250 mM LiCl, 0.5% NP-40, 0.5% sodium deoxycholate, 1 mM EDTA), and 1× TE (10 mM Tris, 1 mM EDTA pH 8.0). After the last wash, 125 µl of elution buffer (TE pH 8.0 with 1% SDS, 150 mM NaCl, and 5 mM dithiothreitol) was added to each sample, and the beads were incubated at 65°C for 10 minutes with a 5 second vortex every 3 minutes. The beads were spun for 2 minutes at 10,000×g, and the supernatant was removed and retained. The elution process was repeated once for a total volume of 250 µl of eluate. For the ChIP input material set aside, elution buffer was added for a total volume of 250 µl. After overlaying the samples with mineral oil, the samples were incubated overnight at 65°C to reverse crosslinks.

### Protein Degradation and DNA Purification

After cooling the samples down to room temperature, each sample was incubated with an equal volume of proteinase K solution (1× TE with 0.4 mg/ml glycogen, and 1 mg/ml proteinase K) at 37°C for 2 hours. Each sample was then extracted twice with an equal volume of phenol and once with an equal volume of 25∶1 chloroform∶isoamyl alcohol. Phase lock gel tubes (Eppendorf) were used to separate the phases (light gel for phenol, heavy gel for chloroform∶isoamyl alcohol). Afterwards, 0.1 volume 3.0 M sodium acetate pH 5.3 and 2.5 volumes of 100% ice cold ethanol were added, and the DNA was allowed to precipitate overnight at −20°C. The DNA was pelleted by centrifugation at 14,000 µg for 15 minutes at 4°C, washed once with cold 70% ethanol, and spun at 14,000 µg for 5 minutes at 4°C. After removing the supernatant, the pellets were allowed to dry and then were resuspended in a in a 30 µl volume supplemented with NEB Buffer 3 (10× concentration of 100 mM NaCl, 50 mM Tris-HCl pH 7.9, 10 mM MgCl_2_, 1 mM dithiothreitol (DTT)) and 0.5 µg of RNase A. The samples were incubated at 37°C for one hour and then treated with 7.5 units of calf intestinal alkaline phosphatase for a further 1 hour at 37°C. Samples were cleaned up with the Qiagen MinElute Reaction Cleanup Kit, following manufacturer's directions, except with an elution volume of 20 µl.

### Linear Amplification of DNA

The samples were amplified, with a starting amount of 125 ng for ChIP input materials and up to 75 ng for ChIP samples, using the DNA linear amplification method described in BMC Genomics 4∶19 and updated with the latest protocol, found here: http://www.broad.harvard.edu/chembio/lab_schreiber/pubs/protocols/IVT_Supplement/supplement.html.

### Microarray Hybridization

3 µg of aRNA produced from the linear amplification were used to label probe via the amino-allyl method as described on www.microarrays.org. Labeled probes were hybridized onto a yeast tiled oligonucleotide microarray [61] at 65°C for 16 hours and washed as described on www.microarrays.org. The arrays were scanned at 5 micron resolution with an Axon Laboratories GenePix 4000B scanner running GenePix 5.1.

### Image Analysis and Data Processing

Array features were filtered using the autoflagging feature. The remaining features for each array were then block normalized by calculating the average net signal intensity for each channel in a given block, and then taking the product of this average and the net signal intensity for each filtered array feature in the block. Afterwards, all block-normalized array features were normalized using a global average net signal intensity as the normalization factor [5]. The median of all replicates was calculated and used for subsequent data analysis.

### Data Availability

All microarray data used in this study have been deposited to GEO, accession #GSE12822. Source code for analysis is available upon request.

### Construction of Turnover Strains

W303-derived MAT**a**, *bar1* strains were used for all these experiments. Wild-type strain PKY3310, as well as *asf1*Δ (PKY3328) and *vps75*Δ (PKY4226) mutant strains were transformed with the plasmid pHHF1-GAL10/1-FLAG-HHT1 [33] to generate PKY4212, PKY4214 and PKY4231, respectively.

### Histone Turnover ChIP

Cell manipulation and chromatin immunoprecipitation were performed essentially as described in Dion *et al.* [33] with slight modification. Briefly, cells were cultured at 30°C in YPARaffinose medium supplemented with Hygromycin B (300 µg/ml). Cells from saturated pre-cultures were inoculated into a final volume of 250 ml, and were maintained in log phase below an OD_600_ of 1.0 for 24 hr. On the following day, cells were adjusted to OD_600_ 0.4 and treated for 3 hrs with α factor (5 µg/ml). Efficient G1 arrest was confirmed by visual inspection for the “shmooed” phenotype, as well as by FACS analysis. To induce expression of the plasmid-borne FLAG-tagged H3, galactose was added to each culture to 0.2%. Forty-five and ninety minutes after galactose addition, cells (100 ml) were harvested by crosslinking for 15 minutes with 1% formaldehyde. The formaldehyde was quenched with glycine (125 mM) for 5 minutes. Subsequent extract preparation, MNase digestion and recovery of immune-complexes were carried out as in Dion et al., 2007 with the following modifications; first, the appropriate amount of MNase needed to digest the chromatin from each strain to 80% mononucleosomes was determined by titration. These amounts varied somewhat from strain to strain, and the chromatin from *asf1*Δ and *vps75*Δ mutants, as compared to wild-type, appeared more resistant to MNase digestion. Additionally, 20 rather than 10 µl of the anti-FLAG antibody was used in immunoprecipitation reactions. DNA amplification and array hybridization were performed as described previously [5].

### Granularization of Microarray Data into Nucleosomes

Microarrays log ratios were granularized into nucleosomal-based data based on nucleosome positions by Yuan *et al.* [61] by taking the median levels per nucleosome [5].

### Preprocessing of Replication Timing Data

The S phase replication time of each nucleosome was calculated based on genomic data by Yabuki *et al.* [44] and Raghuraman *et al.* [43]. The exact time for each nucleosome was linearly interpolated between specified loci/times (0.5 and 1 Kb segments per Raghuraman et al., and Yabuki *et al.*, respectively). To account for different arrest methods and growth conditions, those replication times were linearly transformed to match our cell cycle duration and phase, using Y = X*1.57+6.72 (where Y denotes our times, and X refers to published data). These parameters were optimized to fit the cell cycle expression data using the model's predictions (see below). Origins with transformed S phase replication time <50 minutes were defined as early. Enrichment test for near-origin nucleosomes were based on 10 nucleosomes before/after the origin.

### Midlog H3K56ac Explained by Replication Timing and RI Turnover Rates

The correspondence of H3K56ac levels to replication times and turnover rates were analyzed using LOWESS fit. The input of the algorithm is a set of nucleosomes, whose X coordinate is determined by either S phase replication time (Figure 1A) or turnover rate (Figure 1B), and its Y coordinate determined by its H3K56ac level. For each X value, the Y values of nearest 500 nucleosomes, weighted by tri-cubic weighs based on their [0,1] normalized distances, is fitted by a 1^st^ degree line. Prior to analyzing the data, the top and bottom 1% outlier were trimmed.

To examine the combined fit to replication times and turnover rates (e.g., Figure 1C,D), we implemented 3D LOWESS by extending the 2D smoothing method. The input of the algorithm is a set of nucleosomes, each with turnover rate (X coordinate), replication time (Y coordinate), and H3K56ac level (Z coordinate). The 3D LOWESS algorithm, analyzes every node along a 150×150 exponentially spaced grid (in XY surface). Each such computation was limited to span 500 nearest nucleosomes, weighted by tri-cubic weighs based on their [0,1] normalized distances to the node position on the XY surface. The Z coordinate of neighboring nucleosomes was fitted by a 2D surface to minimize the root mean square deviation, and the node assigned the interpolated Z coordinate. Prior to analyzing the data, the top and bottom 1% outliers were trimmed.

To compute the percent of variance explained by the LOWESS fit, each nucleosome was assigned a “fitted” value F_i_ (linear interpolation of the LOWESS fitting line or surface). Then the variance of the residuals σ^2^_R_ = var({F_I_−Z_i_}) was compared to the variance of the original data σ^2^_D_ = var({Z_i_}). The percent of variance explained (PVE) is defined as 100−100 * (σ^2^_R_/σ^2^_D_).

### From Ratios to Absolute Acetylation Levels

To translate the log ratio measurements into absolute acetylation levels, we modeled the hybridization process. For the midlog data, the actual ratios R_n_ measured by the microarray for nucleosome n, equals log_2_(X_n_/Occ_n_)+C, where X_n_ denotes the percent acetylation of nucleosome n at midlog, Occ_n_ denotes the relative occupation of nucleosome n, and C is an unknown global array normalization factor. The acetylation level can be therefore computed as X̂_n_ = 2^Rn^* Occ_n_* 2^−C^. It should also be noted that X̂_n_ is constrained–it cannot exceed 100% or be less than 0%. We exploited these limits and calibrate the data, by setting the 1^st^ and 99^th^ percentiles at 10% and 90% acetylation levels respectively. The acetylation levels of remaining nucleosomes were linearly interpolated (Figure S6).

We used similar calculations to estimate absolute acetylation levels during the cell cycle. Here, each time point t was compared to midlog H3K56ac levels. Thus, the measured ratio R_n,t_ for nucleosome n at time point t, equals log_2_ X_n,t_/X_n_+C_t_, where X_n_ and X_n,t_ denote the percent acetylation of nucleosome n at midlog or time point t, respectively. We then zero-normalize each nucleosome, and so the normalized ratio M_n,t_ = log_2_ X_n,t_−log_2_ X_n_+C_t_−Z_n_, where the nucleosome-specific normalization Z_n_ equals the temporal average over M_n,t_. More precisely, Z_n_ = E_t_ [log_2_ X_n,t_/X_n_]+E_t_ [C_t_]. Therefore,
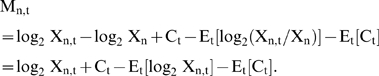
Thus,
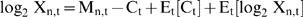
and
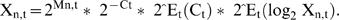



We approximate the absolute acetylation level for each time point using the midlog estimations X̂_n_,
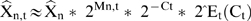



To apply this equation we need the value of array specific normalization factors C_t_. We estimate these based on the bulk acetylation levels for each time point t. This is done in two stages. First we estimate the total amount of nucleosomes for each time point. Second, we estimate the amount of DNA isolated from immunoprecipitated nucleosomes (TLAD) for each time point. By dividing the TLAD-based absolute acetylation levels by the number of nucleosomes, we approximate the bulk H3K56ac levels per time point (Figure S9).

We estimated DNA copy number (up to a global constant) based on S phase replication timing. Each nucleosome was “doubled” at its locus-specific replication timing (assigned as described above). Then, the total number of nucleosome (per minute) was summed. Finally, we simulated the desynchronization of our yeast cultures. This was done using standard models (assuming cell cycle in population rates follow a normal distribution, with mean duration of T = 110 min, and std of 0.09. T) [62],[63].

The amount of H3K56ac nucleosomes for each time point was estimated by fitting the TLAD yields. This was done using a simple Fourier decomposition algorithm, based on the PNM algorithm [62]. The TLAD yields were fitted as a sum of K = 3 sine waves, of periodicities (T/2, T, and 2T), where T equals cell cycle period (110 min). These sine waves were then desynchronized (as above) to account for gradual loss of synchrony. This model has 2K+1 = 7 parameters–each wave is described as the (weighted) sum of pure sine and cosine waves, plus an additional constant. These parameters were optimized to fit the TLAD yields, using a multiple linear regression approach.

Finally, we divided the estimated number of acetylated nucleosomes for each time t (based on the TLAD fit) by the estimated copy number of DNA, to obtain the percent H3K56ac among bulk nucleosomes (Figure S9).

### The H3K56ac Kinetic Model

We developed a simplified model of H3K56ac profiles along the cell cycle. This model simulates the percent acetylation levels of each nucleosome as a non-homogenous Poisson process, where H3K56ac events occur either at the S phase replication time of each nucleosome, or constantly, according to its replication-independent turnover rate. H3K56deac events occur according to the global activity levels of H3K56 deacetylases Hst3/4 (which depend on time). Our model consists of the following parameters:

- K56ac_n_(t)–The estimated number of H3K56 acetylated nucleosomes positioned at locus n.
- K56deac_n_(t)–The estimated number of H3K56 deacetylated nucleosomes positioned at locus n.
- *λ_D_(t)*–a global parameter, shared for all nucleosomes, representing the deacetylation rates by Hst3/4. These rates were tested for a wide range of values, then optimized to a basal rate of deacetylation event of 0.0158 (once every 63 minute, per nucleosome, on average) for the most of the cell cycle phases, and a high rate of 0.1287 (once every 7.8 minutes, per nucleosome, on average) at peak activity of Hst3/4, optimized around 81 minutes (from 79% to 83% of our ∼110 minute cell cycle).
- λ_n_–The replication-independent turnover rate of nucleosomes at locus n. According to the model, such events are accompanied by the genomic incorporation of H3K56ac nucleosome.
- T_n_–The S phase replication timing of each nucleosome. According to the model, at time point T_n_, the DNA at that locus is being replicated. One chromatid holds the old nucleosome (either H3K56ac or H3K56deac), whereas a novel H3K56ac nucleosome is incorporated into the sister chromatid at that same locus. Specifically, this means that the total number of nucleosomes at locus n is double, while the number of H3K56deac nucleosomes remains:
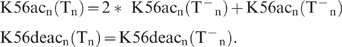

- I_n_–The percentage of nucleosomes (at locus n) acetylated during initial G1 arrest. These parameters were optimized (for each nucleosome) to fit its cell cycle measured log ratios (see below).
- σ_D_–Global desynchronization rate of culture. As described before, cell cycle duration in population rates follow a normal distribution N(T, σT), with T = 110 min and σ = 0.09.

### Master Equation

Apart from T_n_, the changes in K56ac_n_(t) depend on the rate of deacetylated nucleosomes K56deac_n_(t), which are replaced at rate λ_n_ by acetylated ones, minus the rate of acetylated nucleosomes K56ac_n_(t), being deacetylated at rate λ_D_(t). Similarly, the changes in K56deac_n_(t) are exactly opposite (as the total number of nucleosome, apart from replication events, is fixed).
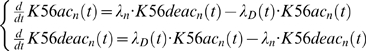



We solved these equations numerically using ODE45 function in MATLAB (R2007a).

### Model-Derived %H3K56ac Profiles

Finally, the percent acetylation of each locus n, at each time t, was calculated by dividing the estimated number of acetylated nucleosomes K56ac_n_(T_n_) by the total number of nucleosomes K56ac_n_(T_n_)+K56deac_n_(T_n_). These percentages were then desynchronized (as described above) to account for natural desynchronization of yeast cultures.

### Fitting the Experimental Data

To fit the cell cycle log ratio measurements of H3K56ac levels, we simulated the hybridization process. Specifically, at each measured time point t, the number of H3K56ac nucleosomes at each loci n, K56ac_n_(t), was divided by the midlog acetylation level (approximated by Σ_t_ K56ac_n_(t)). These ratio were log_2_-transformed, and then normalized (over all genomic loci) to mean zero. Then, extreme ratios were quenched by the symmetric sigmoid function y = 8/(1+e^−x/2^)−4, which is mostly linear for log ratios in [−1.5, 1.5] and asymptotically saturates at −4 and 4. Finally, each nucleosome was zero centered, to match measured data, and the root mean square deviation (RMSD) over all nucleosomes along the entire cell cycle was calculated.

### Optimization

Parameters were optimized to minimize the RMSD between the model predicted profiles and the measured experimental data. First we optimized the few global parameters (the deacetylation rates and phases that determine *λ_D_(t)*, the global desynchronization rate σ_D_). For each combination of 〈onset timing of peak Hst3/4 levels during G2/M; end timing of peak Hst3/4 levels during G2/M; basal level of H3K56deac rate by Hst3/4; and peak level of H3K56deac rate by Hst3/4〉, nucleosome-wise parameters were optimized to achieve the best fit between the predictions of our kinetic model and the actual values. The minimal RMSD was achieved at a basal deacetylation rate of 0.0158 (min^−1^) with peak activity of rate 0.1287 (min^−1^) around the 81^st^ minute of the ∼110 min-long cell cycle. We then optimized the initial acetylation I_n_ at G1 arrest (t = 0) for each nucleosome, as well as its exact S phase replication timing T_n_). This was done using an optimization algorithm for finding the minimum value of unconstrained multivariable functions using a derivative-free method (the fminsearch function in MATLAB, R2007a), with regard to the RMSD of predicted measurements to the actual values loaded. The optimized S phase replication times are well correlated with the measured ones, with Pearson correlation coefficient of 0.78 (p<1e-300).

### Estimation of Turnover Rates Based on Time Series Flag/Nuc Measurment

Replication-independent turnover rates were estimated from a series of arrays measuring the Flag/Nuc ratios along time. We used the algorithm described in Dion *et al.* [33], with the minimal adjustment of replacing the original Flag/Myc ratios R_l_(t) = F(t)/M(t) with Flag/Nuc ratios (as measured), using R_l_(t) = F(t)/(M(t)+F(t)).

## Supporting Information

Figure S1 Average midlog H3K56ac levels for nucleosomes, according to their genomic annotations [5]. Intergenic nucleosomes were assigned to the following categories: Promoter region (anything upstream of a coding region), nucleosome immediately upstream to the TSS (“distal”), and the nucleosome immediately downstream of the TSS (“proximal”). Transcribed regions were separated into 5′ (“AUG”), middle (“CDS”), and 3′ (“STOP”) coding sequences. ARS and tRNA are self-explanatory, and Null refers to any other intergenic region (largely between convergently transcribed genes).
(0.14 MB TIF)

Figure S2 (A) Smoothed H3K56ac levels from midlog cultures (same as Figure 1C) are plotted as a topographical surface. (B) Smoothed H3K56ac levels from G1 arrested cells (same as Figure 1D) are plotted as a topographical surface.
(0.85 MB TIF)

Figure S3 Gene expression levels during cell cycle. Shown are the expression levels from Spellman et al., and two replicates from this study, for 800 periodic genes, sorted by their cell cycle phase [60].
(2.60 MB TIF)

Figure S4 H3K56ac levels along the cell cycle are shown, sorted by (A) replication timing [43],[44], from early (top) to late (bottom), or by (B) G1-arrest RI turnover rates [33], from rapid (top) to slow (bottom) replacements rates.
(3.97 MB TIF)

Figure S5 (A) H3K56ac profiling during the cell cycle, as in Figure 2B. (B) Genomic region near ARS305 shows high early S phase H3K56ac levels, accompanied by low midlog H3K56ac and slow turnover rates. (C) In contrast, H3K56ac levels at nucleosomes around ARS307 peak at G1 and early S phase, accompanied by high midlog H3K56ac levels and rapid turnover rates.
(2.47 MB TIF)

Figure S6 Absolute H3K56ac levels at midlog were reverse engineered based on the measured log ratios for each nucleosome at midlog phase. Analysis of these absolute levels suggest that 31.4% of the bulk population of nucleosomes are H3K56 acetylated. Independent measurements of bulk H3K56ac levels estimated a similar percent (28%) using mass spectrometry [15].
(0.28 MB TIF)

Figure S7 Bulk H3K56ac levels per time point were estimated by comparing the amount of DNA isolated from H3K56ac immunoprecipitated nucleosomes (ChIP yield), to the estimated total number of nucleosomes at each time point. Shown are the ChIP yields (red asterisks), their cell cycle fit, using a desynchronized Fourier decomposition (red line, Methods), an estimation of the bulk DNA copy number (using genome-wide S phase replication times, then desynchronized to match measured data, green line, Methods), and their ratios, which reflect the %H3K56ac profiles along cell cycle (blue).
(0.31 MB TIF)

Figure S8 Simulation of cell cycle and midlog H3K56ac levels. (A) Late replicating nucleosomes with low RI turnover rates (green line) are deacetylated throughout the cell cycle, apart for a short duration from late S phase (due to replication-coupled incorporation of H3K56ac nucleosome) to G2 phases (peak activity of deacetylases Hst3/4). Alternatively, early replicating nucleosomes with low RI turnover rates (black), are also deacetylated throughout the cell cycle, but are acetylated for a longer period–from early S to G2 phase. Finally, nucleosomes with rapid RI turnover rates (red line) are acetylated throughout the cell cycle (due to replication-independent turnover events), and are show lower acetylation levels when the activity of Hst3/4 peaks around G2 phase (B) For midlog cultures, the acetylation profiles described in (A) are averaged over unsynchronized population, resulting with low H3K56ac levels for cold/late nucleosomes, and high H3K56ac levels for hot/early nucleosomes.
(0.40 MB TIF)

Figure S9 Comparison of the measured S phase replication times (linearly transformed from [43],[44]), and the ones optimized by our kinetic model to fit the cell cycle H3K56ac profiles. The replication times are well correlated, with Pearson correlation coefficient of 0.78 (p<1e-300).
(0.24 MB TIF)

Figure S10 Western blotting anti-H3K56ac antibody characterization: (A) 0.2 OD cell equivalents of protein, extracted by alkaline lysis, were resolved in a 15% Anderson gel, transferred to nitrocellulose and blotted with an anti-K56ac H3 antibody (Upstate, 1∶5000). (B) Upstate H3K56ac antibody (1∶5000) was tested for specificity by spot blotting of the indicated peptides (2, 10, 25 and 50 pmol). Where indicated, the antibody solution was pre-absorbed with the non-acetylated peptide (0.05 mg/ml) prior to blotting.
(0.46 MB TIF)

Table S1 K56ac antibody ChIP yields from wt, K56R, and *rtt109*Δ yeast.
(0.03 MB XLS)

Dataset S1 H3K56ac levels during Midlog and Cell Cycle.
(0.25 MB XLS)

Dataset S2 Replication-independent turnover rates during G1 and M phases, mutant strains.
(3.24 MB XLS)

Dataset S3 Enriched annotations for nucleosomes with rapid G1 or M phase turnover rates.
(0.23 MB ZIP)
