# Supplementary figures and images for: Cell Cycle– and Chaperone-Mediated Regulation of H3K56ac Incorporation in Yeast
Source: PLoS Genet. 2008 Nov 21;4(11):e1000270. doi: 10.1371/journal.pgen.1000270 (PMC2581598; doi:10.1371/journal.pgen.1000270)

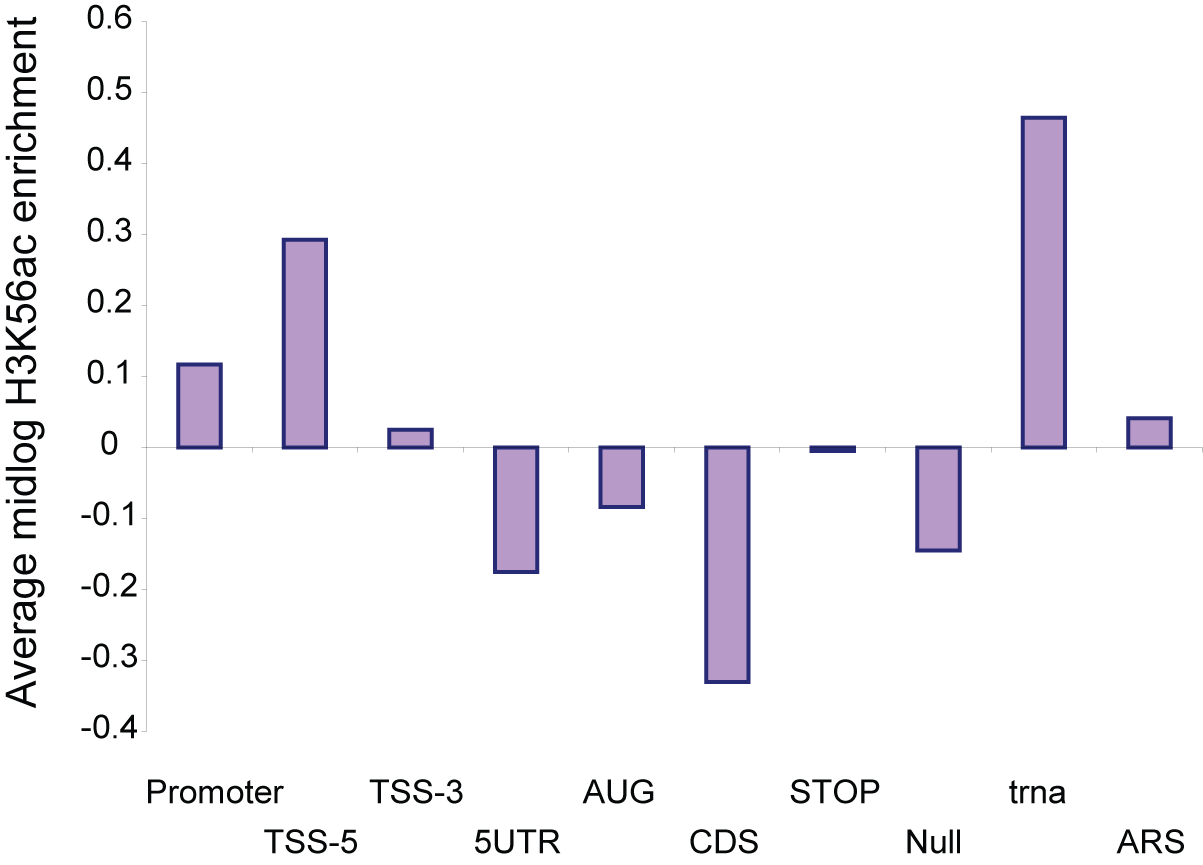

Supplement: Figure S1 — Average midlog H3K56ac levels for nucleosomes, according to their genomic annotations [5]. Intergenic nucleosomes were assigned to the following categories: Promoter region (anything upstream of a coding region), nucleosome immediately upstream to the TSS (“distal”), and the nucleosome immediately downstream of the TSS (“proximal”). Transcribed regions were separated into 5′ (“AUG”), middle (“CDS”), and 3′ (“STOP”) coding sequences. ARS and tRNA are self-explanatory, and Null refers to any other intergenic region (largely between convergently transcribed genes). (0.14 MB TIF) [file pgen.1000270.s001.tif]

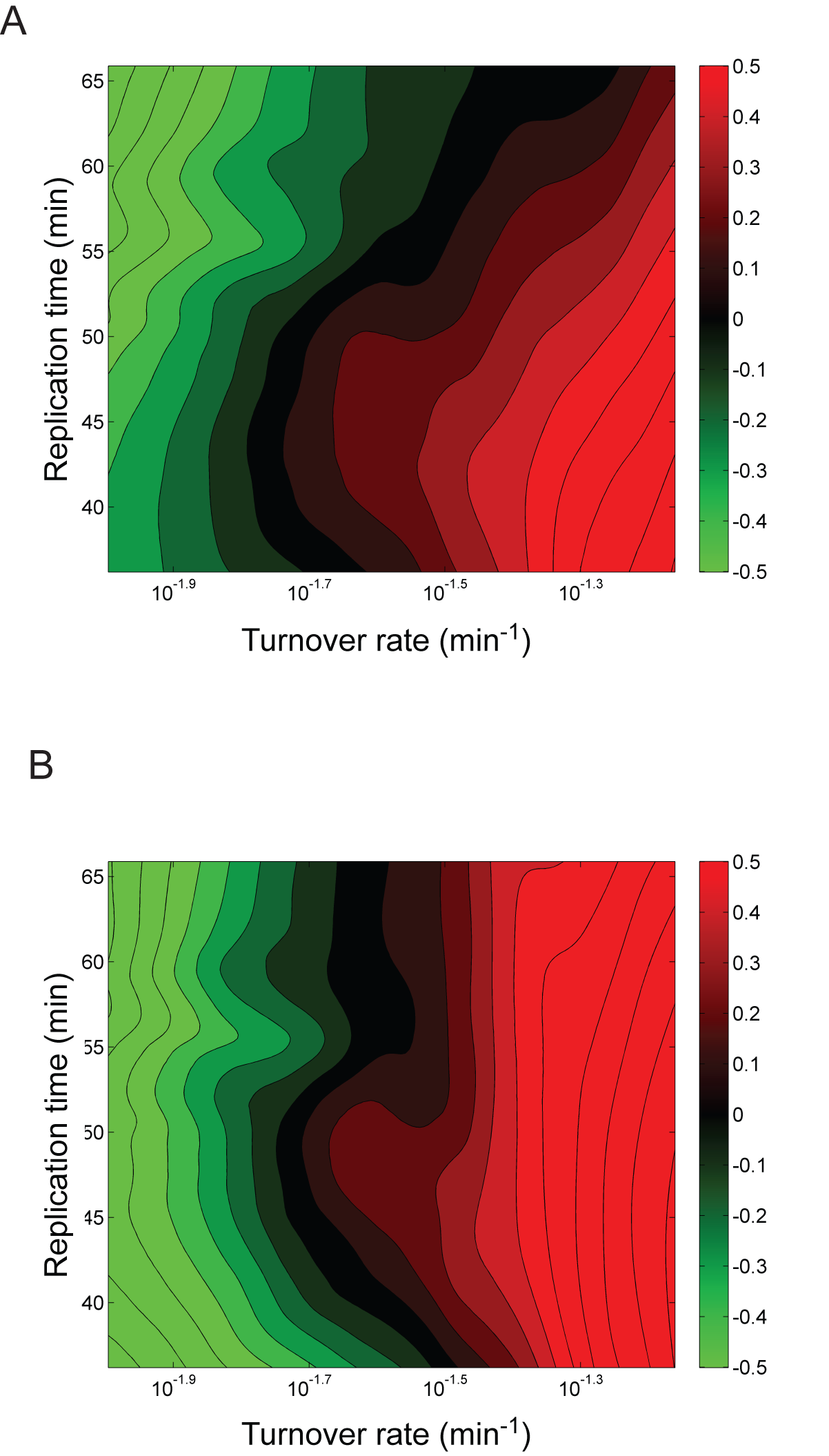

Supplement: Figure S2 — (A) Smoothed H3K56ac levels from midlog cultures (same as Figure 1C) are plotted as a topographical surface. (B) Smoothed H3K56ac levels from G1 arrested cells (same as Figure 1D) are plotted as a topographical surface. (0.85 MB TIF) [file pgen.1000270.s002.tif]

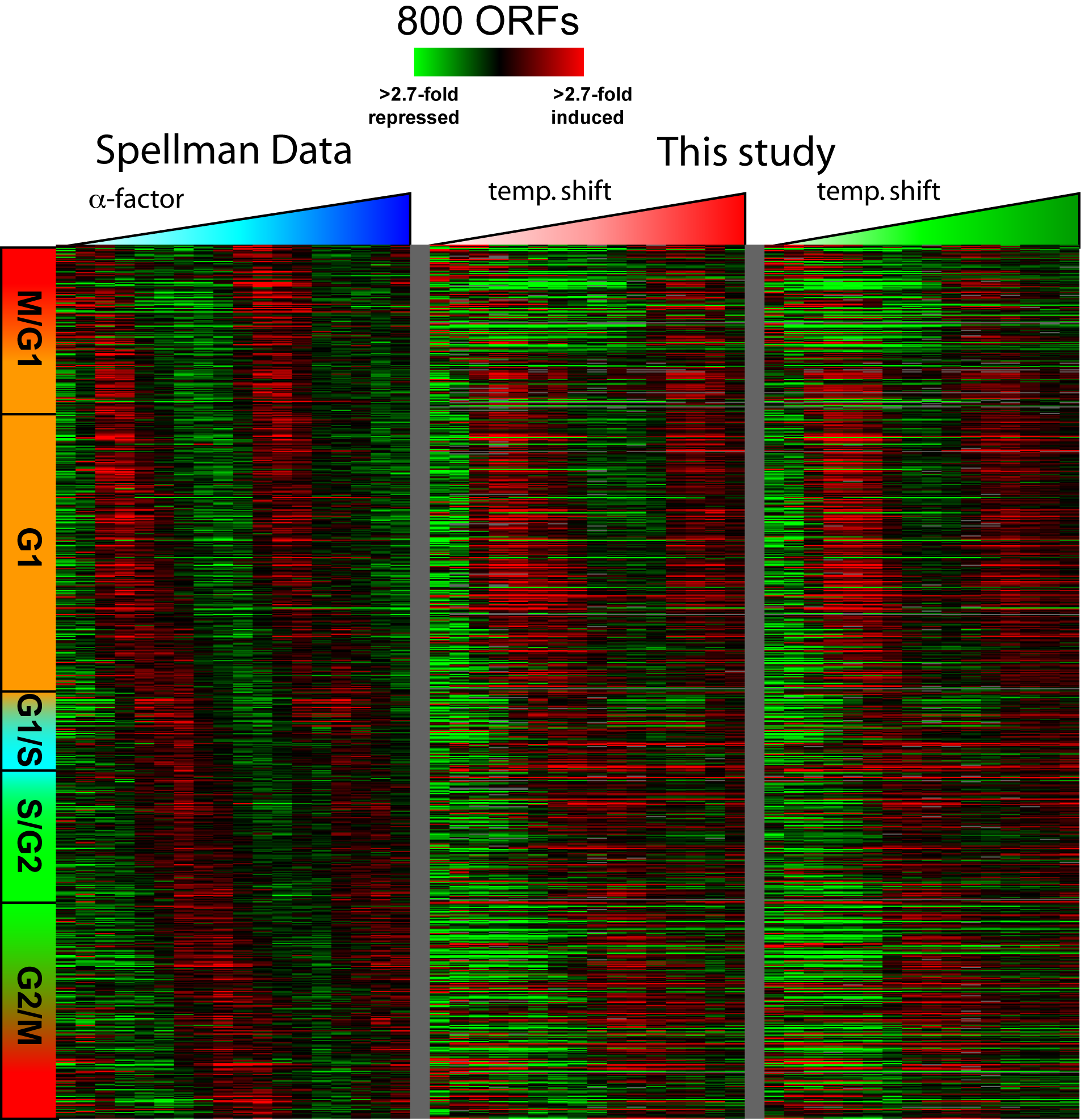

Supplement: Figure S3 — Gene expression levels during cell cycle. Shown are the expression levels from Spellman et al., and two replicates from this study, for 800 periodic genes, sorted by their cell cycle phase [60]. (2.60 MB TIF) [file pgen.1000270.s003.tif]

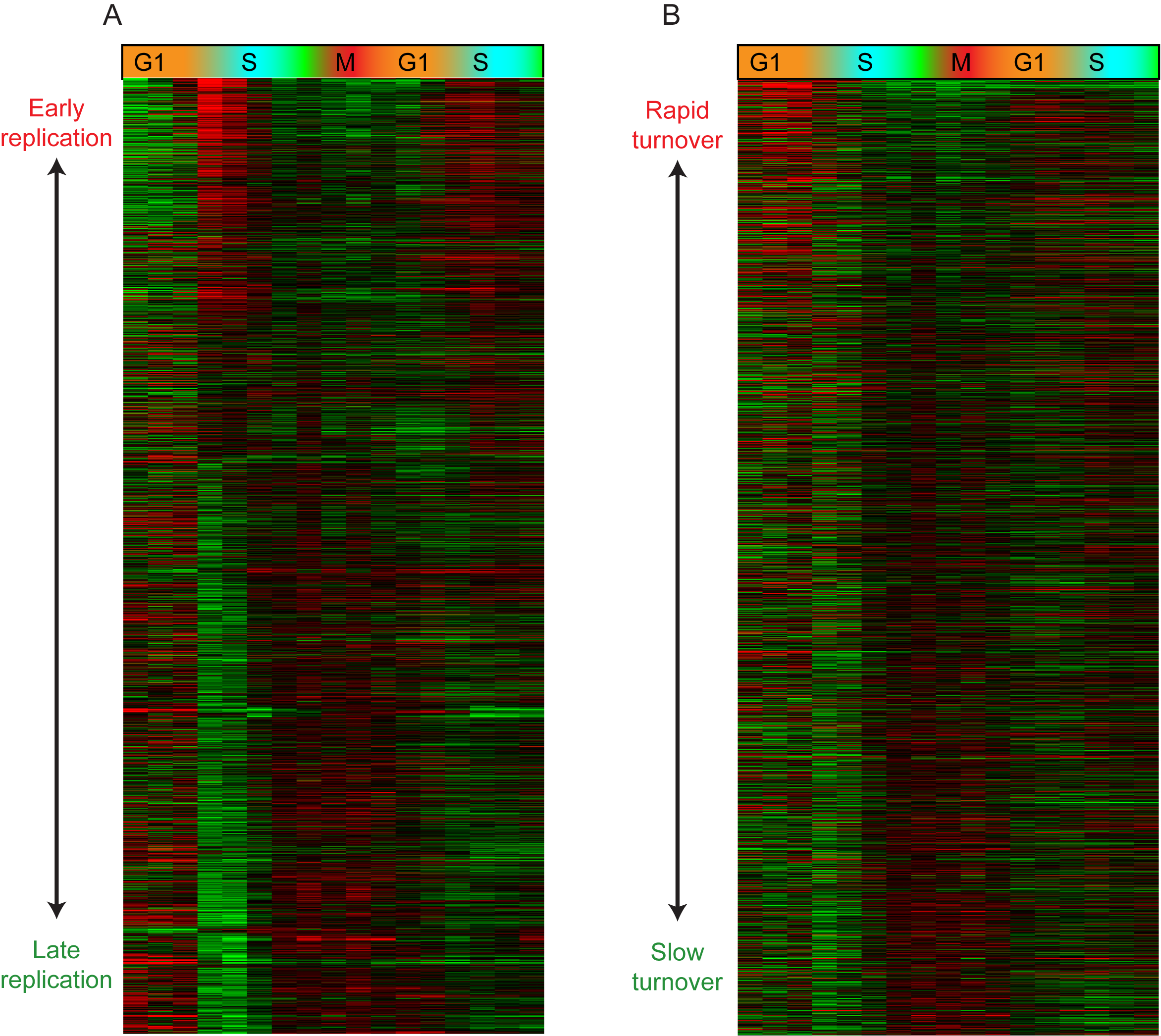

Supplement: Figure S4 — H3K56ac levels along the cell cycle are shown, sorted by (A) replication timing [43],[44], from early (top) to late (bottom), or by (B) G1-arrest RI turnover rates [33], from rapid (top) to slow (bottom) replacements rates. (3.97 MB TIF) [file pgen.1000270.s004.tif]

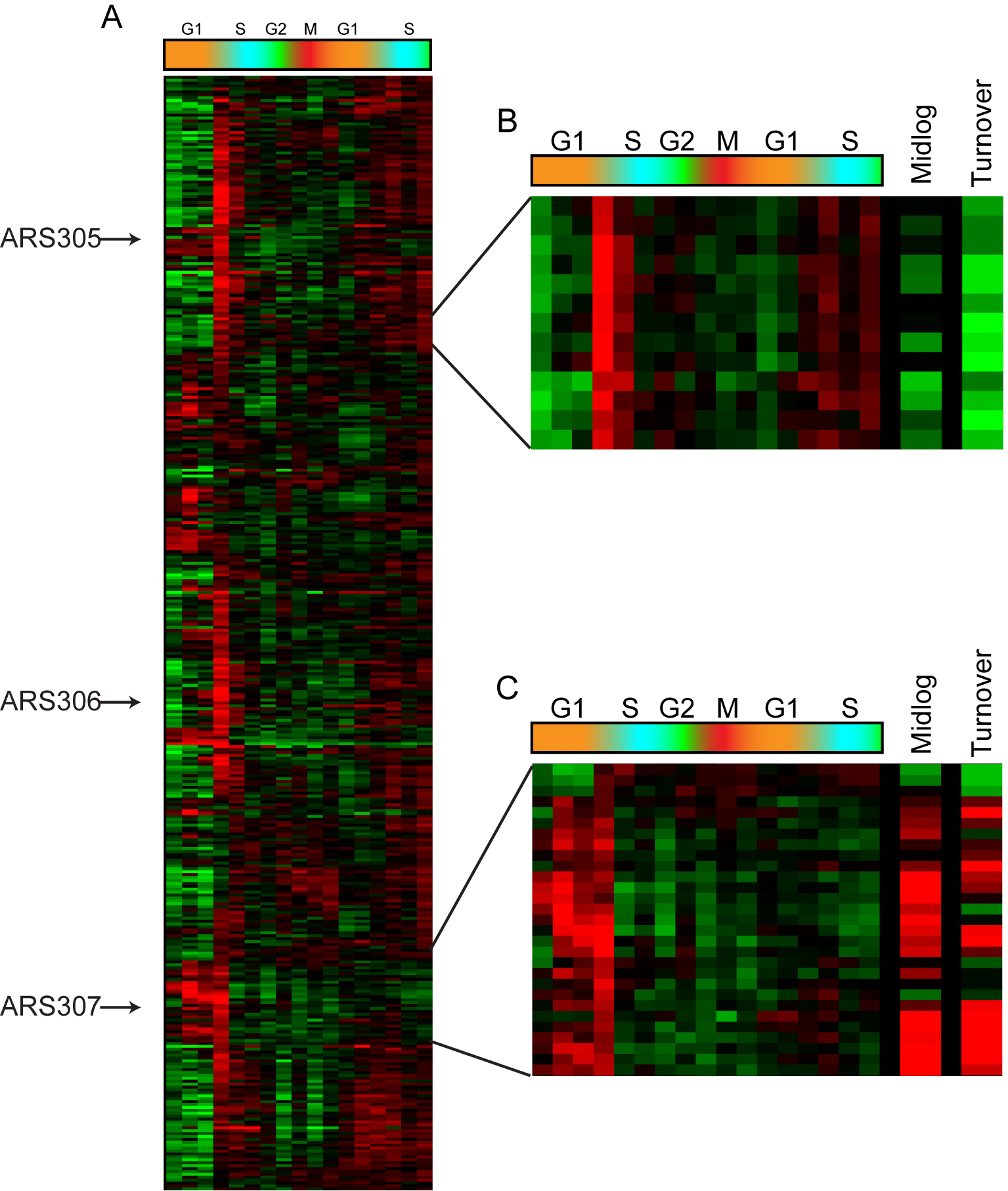

Supplement: Figure S5 — (A) H3K56ac profiling during the cell cycle, as in Figure 2B. (B) Genomic region near ARS305 shows high early S phase H3K56ac levels, accompanied by low midlog H3K56ac and slow turnover rates. (C) In contrast, H3K56ac levels at nucleosomes around ARS307 peak at G1 and early S phase, accompanied by high midlog H3K56ac levels and rapid turnover rates. (2.47 MB TIF) [file pgen.1000270.s005.tif]

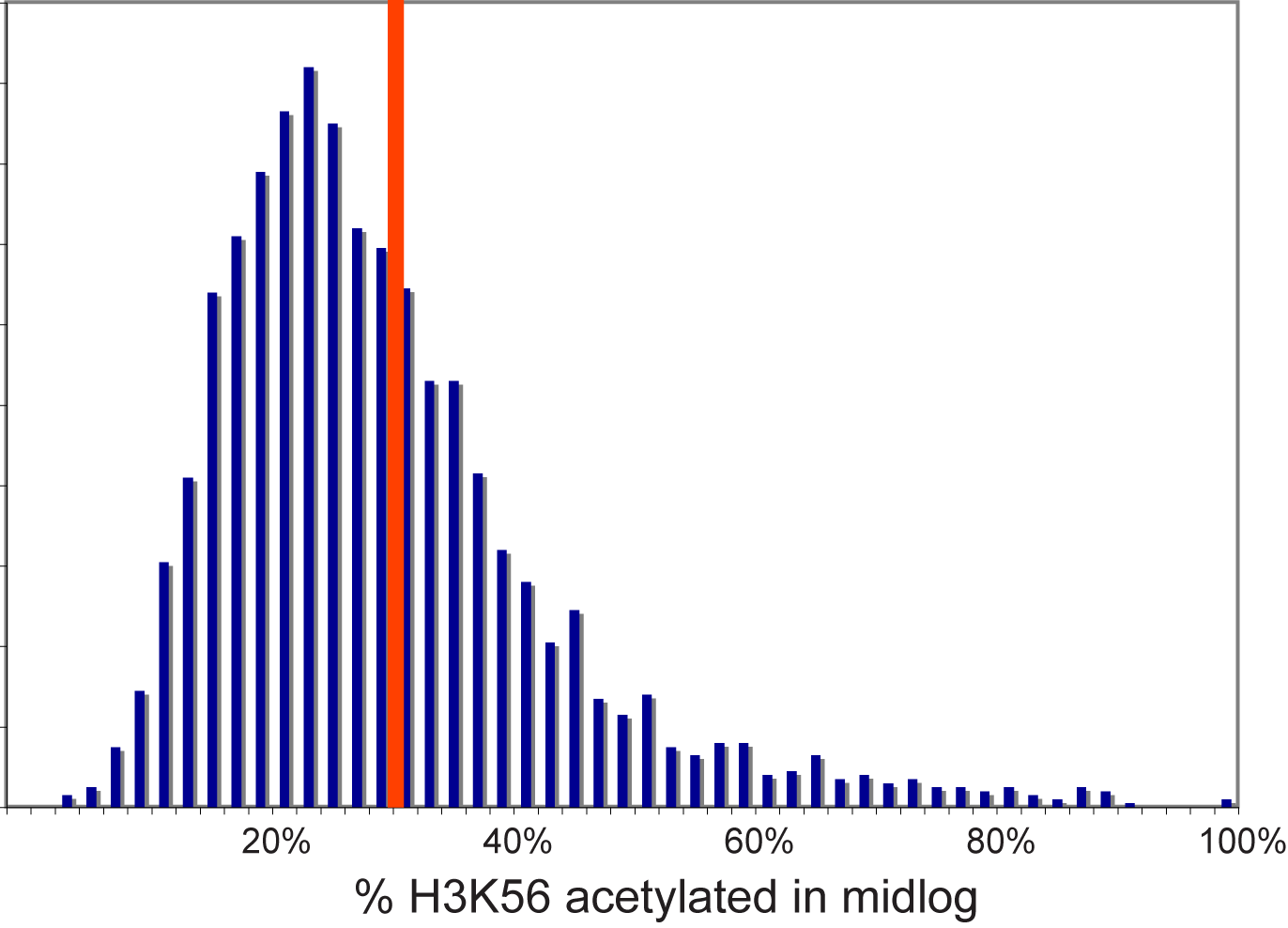

Supplement: Figure S6 — Absolute H3K56ac levels at midlog were reverse engineered based on the measured log ratios for each nucleosome at midlog phase. Analysis of these absolute levels suggest that 31.4% of the bulk population of nucleosomes are H3K56 acetylated. Independent measurements of bulk H3K56ac levels estimated a similar percent (28%) using mass spectrometry [15]. (0.28 MB TIF) [file pgen.1000270.s006.tif]

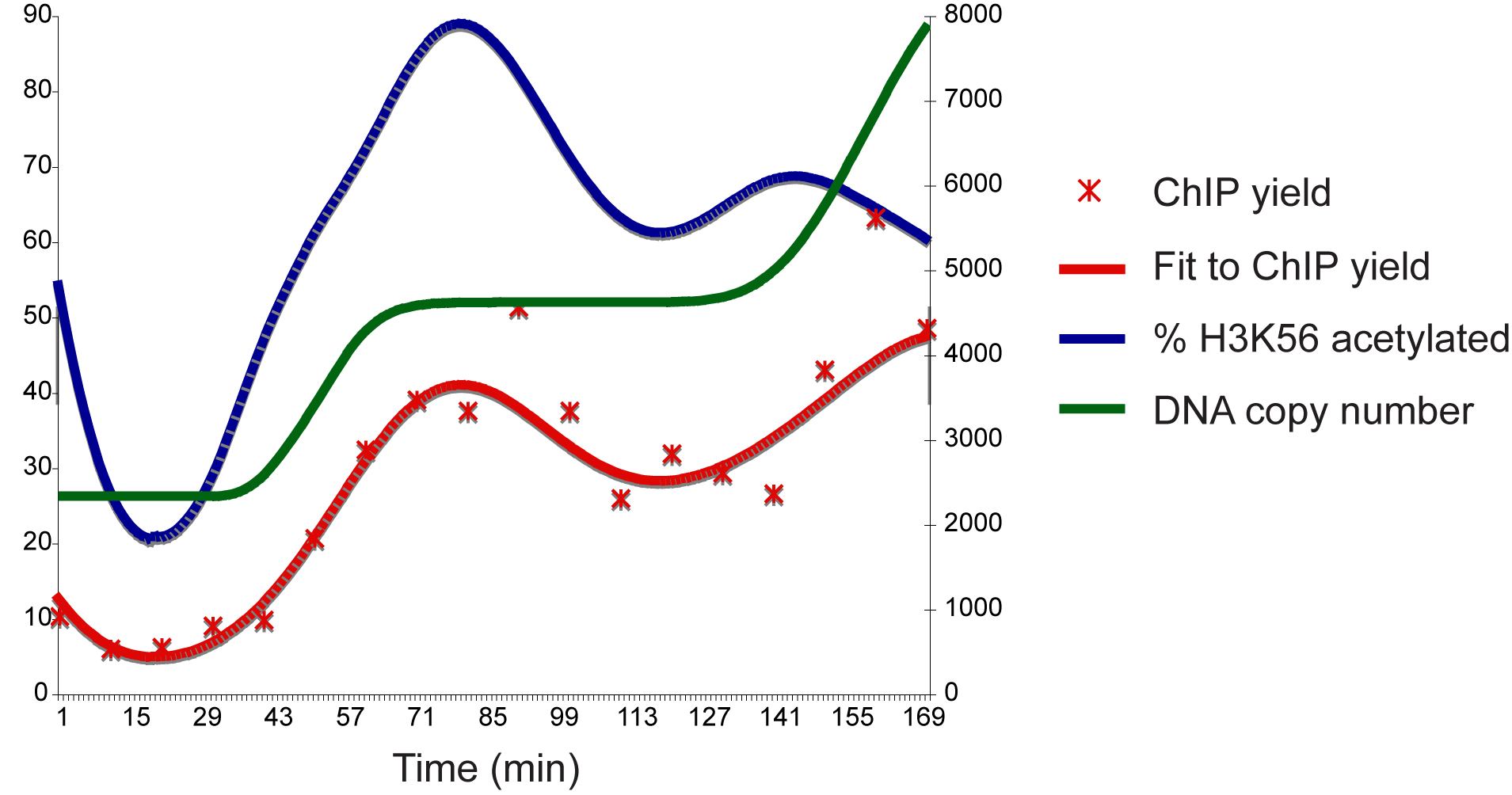

Supplement: Figure S7 — Bulk H3K56ac levels per time point were estimated by comparing the amount of DNA isolated from H3K56ac immunoprecipitated nucleosomes (ChIP yield), to the estimated total number of nucleosomes at each time point. Shown are the ChIP yields (red asterisks), their cell cycle fit, using a desynchronized Fourier decomposition (red line, Methods), an estimation of the bulk DNA copy number (using genome-wide S phase replication times, then desynchronized to match measured data, green line, Methods), and their ratios, which reflect the %H3K56ac profiles along cell cycle (blue). (0.31 MB TIF) [file pgen.1000270.s007.tif]

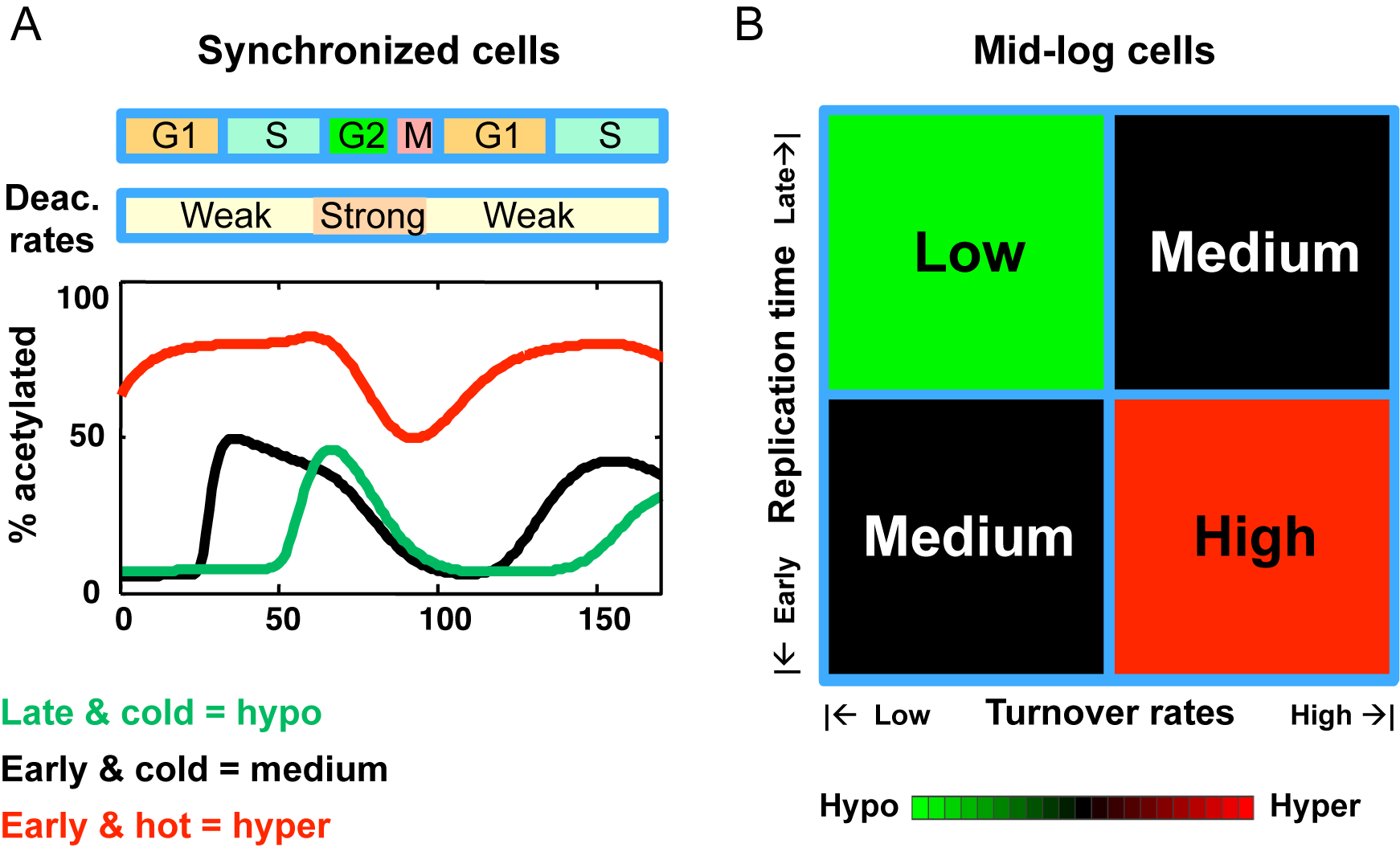

Supplement: Figure S8 — Simulation of cell cycle and midlog H3K56ac levels. (A) Late replicating nucleosomes with low RI turnover rates (green line) are deacetylated throughout the cell cycle, apart for a short duration from late S phase (due to replication-coupled incorporation of H3K56ac nucleosome) to G2 phases (peak activity of deacetylases Hst3/4). Alternatively, early replicating nucleosomes with low RI turnover rates (black), are also deacetylated throughout the cell cycle, but are acetylated for a longer period–from early S to G2 phase. Finally, nucleosomes with rapid RI turnover rates (red line) are acetylated throughout the cell cycle (due to replication-independent turnover events), and are show lower acetylation levels when the activity of Hst3/4 peaks around G2 phase (B) For midlog cultures, the acetylation profiles described in (A) are averaged over unsynchronized population, resulting with low H3K56ac levels for cold/late nucleosomes, and high H3K56ac levels for hot/early nucleosomes. (0.40 MB TIF) [file pgen.1000270.s008.tif]

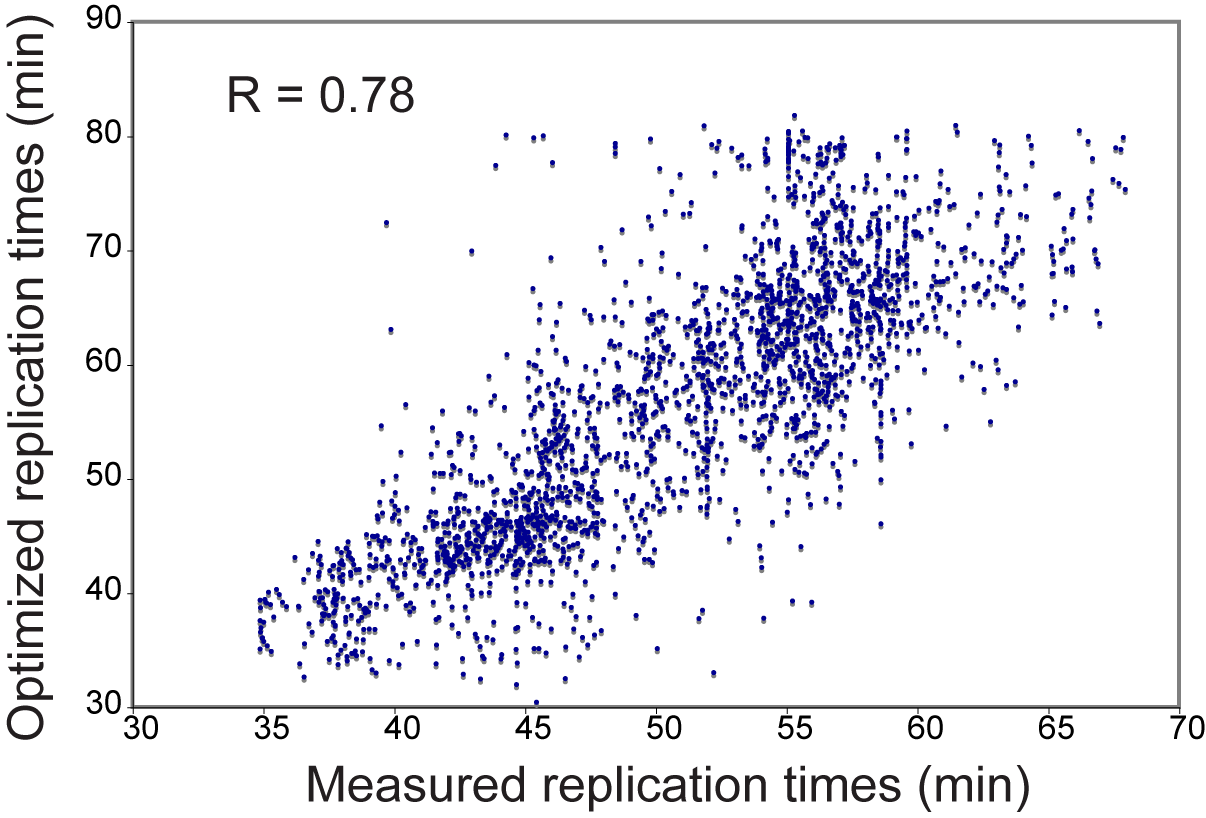

Supplement: Figure S9 — Comparison of the measured S phase replication times (linearly transformed from [43],[44]), and the ones optimized by our kinetic model to fit the cell cycle H3K56ac profiles. The replication times are well correlated, with Pearson correlation coefficient of 0.78 (p<1e-300). (0.24 MB TIF) [file pgen.1000270.s009.tif]

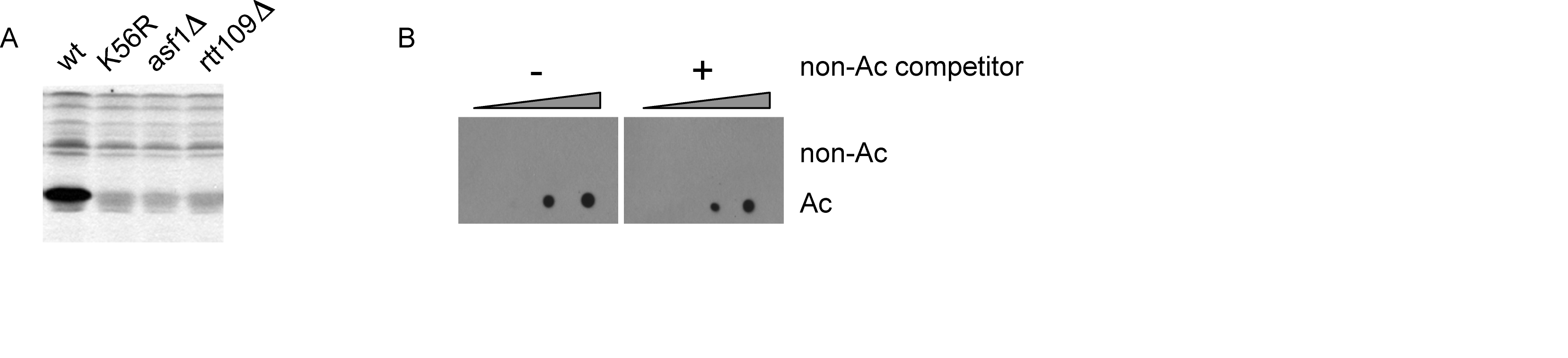

Supplement: Figure S10 — Western blotting anti-H3K56ac antibody characterization: (A) 0.2 OD cell equivalents of protein, extracted by alkaline lysis, were resolved in a 15% Anderson gel, transferred to nitrocellulose and blotted with an anti-K56ac H3 antibody (Upstate, 1∶5000). (B) Upstate H3K56ac antibody (1∶5000) was tested for specificity by spot blotting of the indicated peptides (2, 10, 25 and 50 pmol). Where indicated, the antibody solution was pre-absorbed with the non-acetylated peptide (0.05 mg/ml) prior to blotting. (0.46 MB TIF) [file pgen.1000270.s010.tif]
